# Helicenes with Four Helical Turns: Dimerization of [13]Helicenes to [27]Helicenoids

**DOI:** 10.1002/anie.202506328

**Published:** 2025-05-27

**Authors:** Matea Sršen, Stephan K. Pedersen, Tomislav Rožić, Arianna Lanza, Michael Pittelkow

**Affiliations:** ^1^ Department of Chemistry University of Copenhagen Universitetsparken 5 Copenhagen Ø DK2100 Denmark

**Keywords:** Chirality, Helicenes, Non‐planar aromatics, Structure–property relationships, Synthetic methods

## Abstract

The promise of enhanced circularly polarized luminescence (CPL) from organic molecules has inspired synthetic efforts to prepare elongated multi‐layer helicenes. It has proven particularly challenging to synthesize and isolate enantiomerically pure multi‐turn helicenes, and consequently, clear guidelines for the molecular design of helicenes to achieve large CPL output remain elusive. We explore the oxidative dimerization of a 2‐naphthol‐annulated hetero[13]helicene, and observe the formation of two types of structurally distinct hetero[27]helicenes, both with four helical turns, form. With Cu^II^Cl(OH)‐TMEDA as the oxidant, a symmetric dimer (**bi[13]**) formed, joining two helicenes with the same helicity in their keto tautomeric forms. Using Cu^II^(OTf)₂ as the oxidant led to an unsymmetrical [27]helicenoid composed by an (*M*)‐ and a (*P*)‐enantiomer of the [13]helicene through an unusual coupling between the 1‐ and 3‐positions of the 2‐naphthol units. Structural characterization was achieved by NMR spectroscopy and single‐crystal (X‐ray or electron) diffraction analysis. The enantiomers of this **[27]helicenoid** were analyzed by electronic circular dichroism (ECD) and CPL measurements. The spectroscopic data were corroborated by DFT calculations, and the intense CPL output is preserved despite the presence of an (*M* )‐ and a ( *p* )‐helicene in the same molecule. These [27]helicenoids are the first isolated structures that feature helical π‐systems with four helical turns.

## Graphical abstract

The telephone cord challenge: When a 2‐naphthol‐annulated hetero[13]helicene is oxidatively dimerized, two structurally distinct hetero[27]helicenes (both with four helical turns) are formed. When a racemic mixture of [13]helicenes is dimerized, a (*M*)‐ and a (*P*)‐helicene join to form a mixed [27]helicenoid (right), and when an enantiomerically pure [13]helicene is dimerized an unusual bis‐keto form of the naphthol units is formed (left).

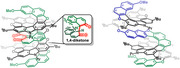

## Introduction

Helicenes are fully conjugated, *ortho*‐fused molecules that are chiral due to conformational constraints.^[^ ^1^, ^2^, ^3^, ^4^, ^5^, ^6^ ^]^ The helical chirality and the associated unique chiroptical properties, such as electronic circular dichroism (ECD) and circularly polarized luminescence (CPL), make helicenes attractive for applications in CLP‐OLEDs.^[^ ^7^, ^8^, ^9^, ^10^, ^11^, ^12^, ^13^, ^14^ ^]^ Recent observations suggest that elongating helicenes and other helical structures can lead to a strong amplification of asymmetry, potentially enhancing chiroptical properties in these molecules.^[^ ^15^, ^16^, ^17^ ^]^ This phenomenon has spurred significant interest in the synthesis of longer helically chiral molecules and materials over the past few decades.^[^ ^15^, ^18^, ^19^, ^20^ ^]^ Polymeric helicene‐like architectures, such as helically coiled graphene nanoribbon, have also emerged as a promising approach to amplify chiroptical and magnetic properties, as demonstrated in recent studies.^[^ ^21^, ^22^, ^23^ ^]^ However, based on the current experimental literature how the chiroptical responses of helicenes, specifically ECD and CPL intensities, scale with the addition of helical turns in a homologous series.^[^ ^24^ ^]^ Computational studies suggest that the ECD response upon elongation of carbo[*n*]helicenes up to *n*  =  30 benzene rings becomes dominated by low‐energy transitions when *n* is larger than 12, corresponding to roughly two helical turns.^[^ ^25^ ^]^ In recent work together with the groups of Nuckolls and Santoro, we demonstrated more‐than‐linear amplification of both ECD and CPL (*g*_lum_) output in elongated perylene diimide (PDI) helicenes as their length increases.^[^ ^26^ ^]^

**Figure 1:**
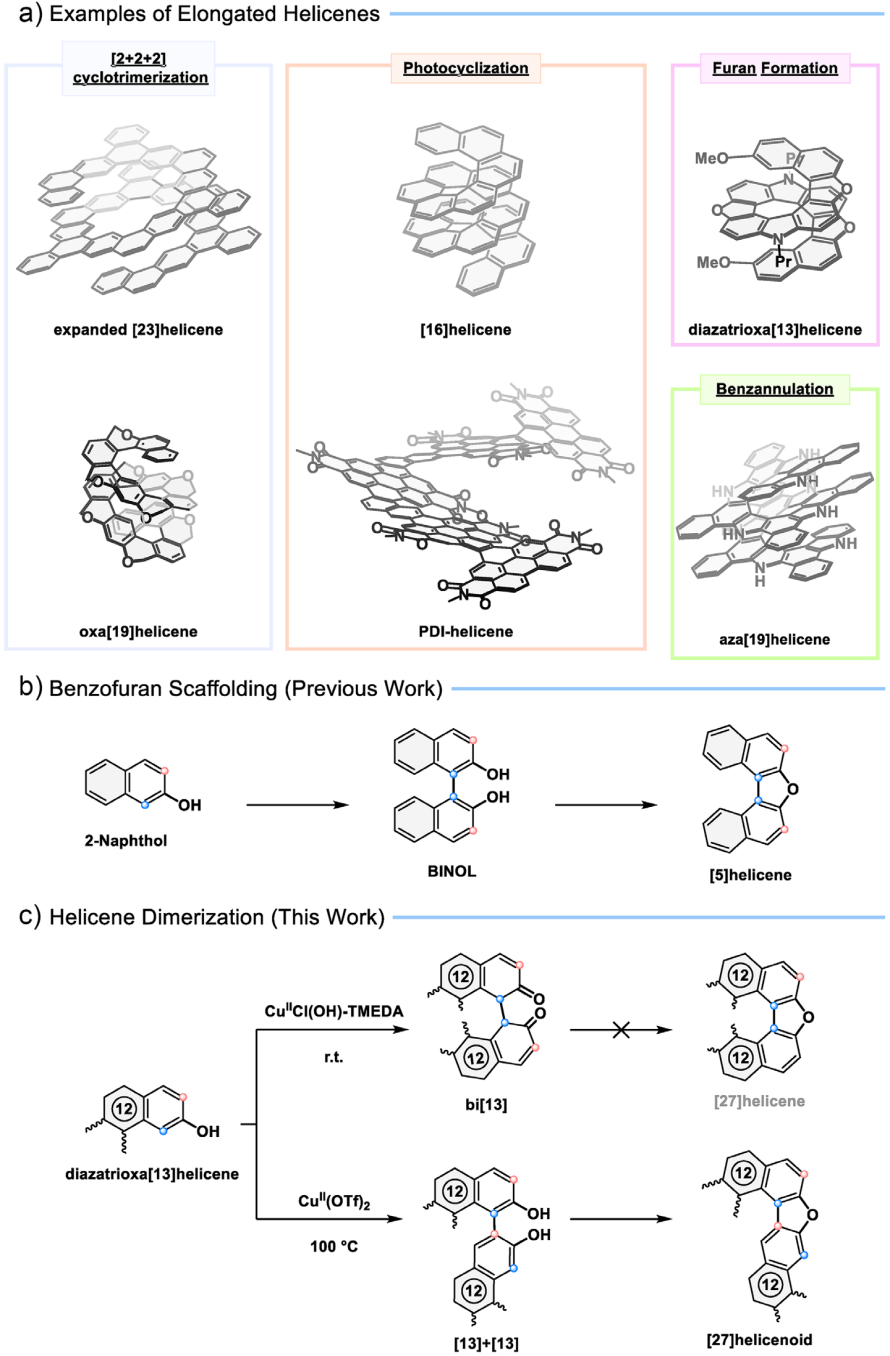
a) A selection of π‐elongated helicenes, and the key synthetic methodology used to synthesize them.^[^ ^29^, ^30^, ^31^, ^32^, ^33^, ^34^ ^]^ b) The benzofuran scaffolding strategy illustrated by the oxidation of 2‐naphthol to BINOL followed by a dehydration reaction to yield a [5]helicene.^[^ ^35^ ^]^ c) Unusual helicenoids (**bi[13]** and **[27]helicenoid**) described in this work. The connectivity in the benzofuran scaffolding is highlighted.

This amplification of both *g*_lum_^[^ ^27^ ^]^ and the fluorescence brightness (*B*_lum_)^[^ ^28^ ^]^ results from the progressive alignment of magnetic (|*m*|) and electric (|*μ*|) transition dipole moment vectors, as the helical backbone gets extended. These results suggest that synthesizing longer helicenes could further amplify chiroptical effects. While structures with one to three helical turns have been reported (Figure 1a),^[^ ^29^, ^30^, ^31^, ^32^, ^33^ ^]^ making multi‐turn helicenes remains a major synthetic challenge that we seek to address in the present work.

In our previous work, we explored a benzofuran scaffolding strategy to elongate helicenes, which has proven to be a powerful transformation en route to different helicenes such as a range of two‐layered diazadioxa[13]helicenes.^[^ ^31^ ^]^ This strategy is straightforward: first, an oxidative C–C coupling at the *ortho* positions of two phenols links the helicene fragments, followed by acid‐mediated dehydration to form a furan ring (Figure 1b). We aimed to further extend this synthetic methodology to prepare longer helicenes by dimerization of diazadioxa[13]helicenes followed by furan ring formation. Here, we observe unexpected reactivity in the annulated 2‐naphthol moieties, namely a coupling between the 1‐ position of one 2‐naphthol and the 3‐position of the other 2‐naphthol moiety (**[27]helicenoid**, Figure 1c). We also observe the trapping of the π‐extended BINOL systems in the di‐keto configuration (**bi[13]** Figure 1c).

## Results and Discussion

### Synthesis

**Scheme 1:**
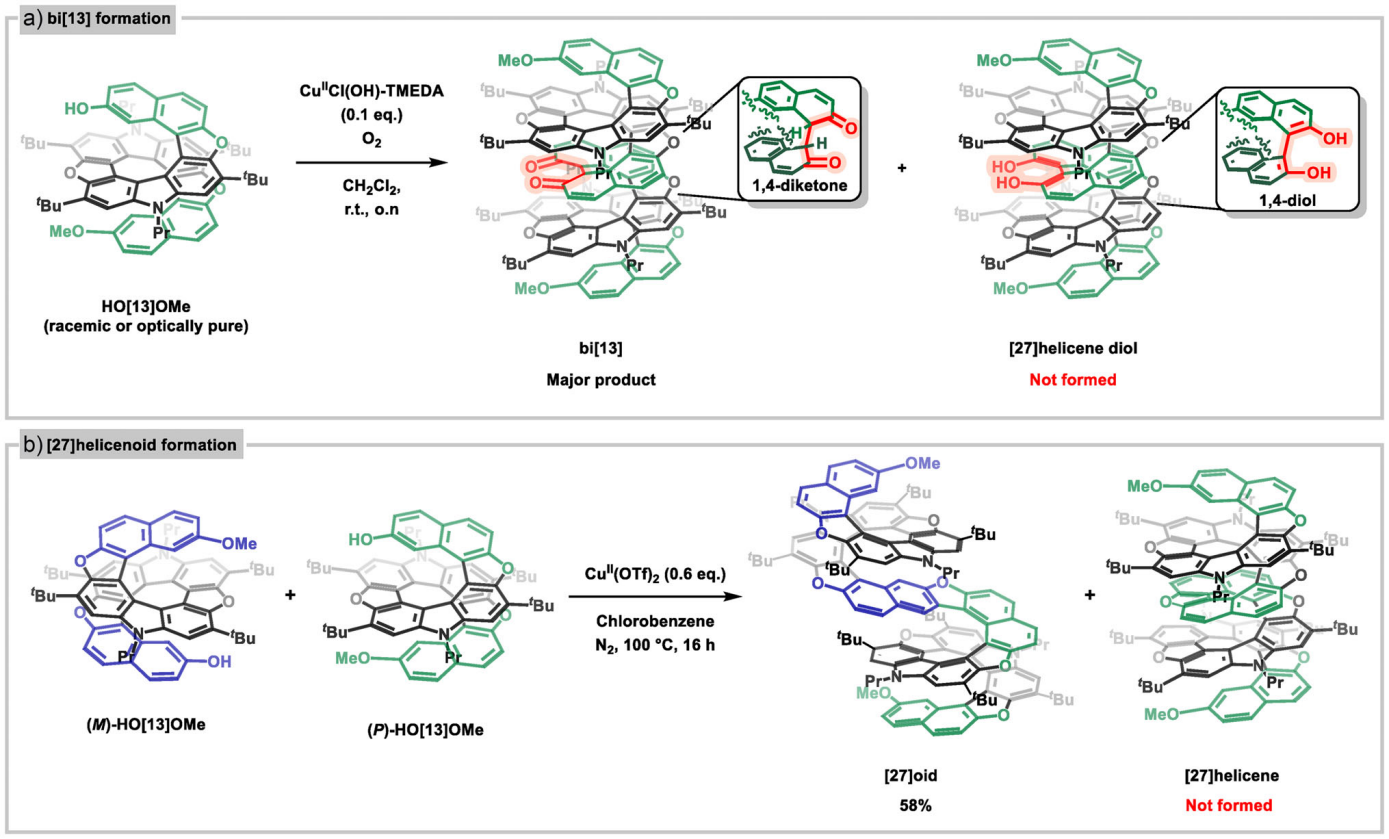
a) Synthesis of **bi[13]** diketone four‐layered helicenoid structure by stereoselective dimerization of either (*m*)‐ or (*p*)‐[13]helicene. b) Synthesis of **[27]helicenoid** by dimerization of an (*m*)‐ and a (*p*)‐[13]helicene with oxidative coupling between a 1‐ and a 3‐position of the 2‐naphthol units.

We prepared the **HO[13]OMe** helicenes according to previously published protocols.^[^ ^31^ ^]^ The extension of the helicene backbones by benzofuran scaffolding approach with 2‐naphthol derivatives proved challenging. We focused our efforts on benzofuran formation by [13]helicene dimerization followed by dehydration. We soon realized that different products were formed when applying the dimerization strategy. The product formation depended on whether a racemic mixture of ( *m* )‐ and ( *p* )‐helicenes or the enantiopure helicene (either ( *m* )‐ or ( *p* )‐helicenes) was used, and also on the applied reaction conditions (Scheme 1). When enantiomerically pure [13]helicene was used as the starting material in combination with Cu^II^Cl(OH)‐TMEDA (TMEDA = tetramethylethylendiamine) as the oxidant, the formation of the 1,4‐diketone dimer **bi[13]** was observed instead of the expected 1,4‐πdiol dimer (Scheme 1a).^[^ ^36^ ^]^ The unusual behavior of phenols to yield the 1,4‐diketones is not entirely without precedents, as similar motifs have been observed by Shinokubo and co‐workers when dimerizing functionalized 2‐hydroxy anthracenes.^[^ ^36^ ^]^ When racemic [13]helicene was used, **bi[13]** remained the major product. Subjecting **bi[13]** to the acidic reaction conditions typically used to form a furan ring—and with that [27]helicene—led to reversion of the **bi[13]** dimer back to the starting material. The reversal of the **bi[13]** to **HO[13]OMe** is another unusual transformation as there is no obvious mechanistic explanation, and yet Tanaka and co‐workers have observed a similar reaction in the attempt to dimerize a chiral pyrrole‐containing helicene.^[^ ^37^ ^]^ They observed that the reversal to monomers could also be mediated with light, but they do not comment on the source of the reducing agent or the source of the additional hydrogen atoms needed. In our reaction, elevated temperatures caused decomposition into an unidentified mixture of products. Treatment with base also resulted in decomposition, and hence is also not a viable route to the [27]helicene. Under these reaction conditions, this set of experiments thus highlights the narcissistic behavior of the reaction, where ( *m* )‐helicenes couple exclusively with ( *m* )‐helicenes and ( *p* )‐helicenes couple exclusively with ( *p* )‐helicenes. The **bi[13]** structure presents an example of a significantly π‐extended naphthol‐keto‐form, and the presence of this structural configuration highlights the challenge of forming strained π‐systems, such as extended helicenes.

When a racemic mixture of **HO[13]OMe** helicene was treated with Cu^II^(OTf)_2_ (Scheme 1b) a benzofuran product was detected with high‐resolution mass spectrometry (HRMS). To our surprise, the isolated strongly blue fluorescent product had lost its symmetry, as observed by analyzing the ^1^H NMR spectrum. In fact, a selective direct oxidative coupling reaction between the 1‐ and 3ʹ‐positions of the two helicenes with opposite helicity had occurred (**[27]helicenoid**). This is surprising for a number or reasons. It is unprecedented to observe coupling in the 3‐position of 2‐naphthols, and the homochiral reactivity observed in the Cu^II^Cl(OH)‐TMEDA ‐mediated reactions is reversed. On the other hand, the structure does gain a larger almost‐planar aromatic region, which may be a driving force. Dehydration was not observed below 100 °C; however, at this elevated temperature, the reaction proceeded to form the furan ring, resulting in the formation of the helicene‐like product in 58% isolated yield.

### Structural analysis

The **bi[13]** compound was found to be unstable during chromatographic purification, predominantly reverting to the starting material, **HO[13]OMe**. Consequently, the NMR spectroscopic characterization was conducted on the crude reaction mixture after work‐up but before (any) chromatographic separation (see Supporting Information; Figure S4).

**Figure 2:**
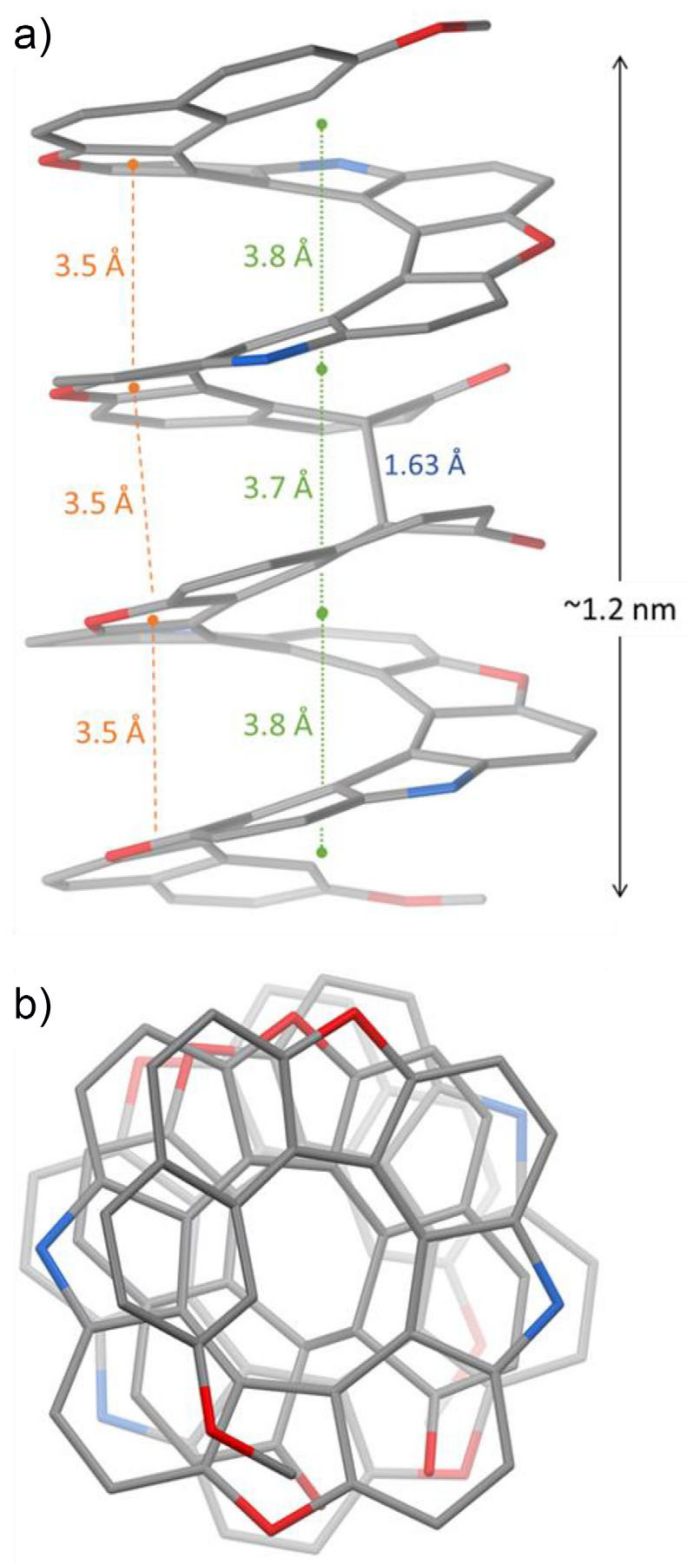
a) Simplified molecular structure of (*P*)‐**bi[13]**. The average distances between consecutive turns are reported in green, while the closest stacking distances, found between furan rings, are shown in orange. The value in blue refers to the central C–C single bond. Color code: C = grey, O = red, N = blue. Alkyl groups and hydrogen atoms are omitted for clarity. See Supporting Information for the distances measurement method. b) The same molecule viewed along the helix axis, showing the four turns.

By observing the ^1^H–^13^C HSQC NMR spectrum of crude **bi[13]** in CD_2_Cl_2_, the sp^3^ hybridized C was detected at 57.0 ppm (Figure S7) with the corresponding proton at 2.63 ppm (Figure S5). We also observe a clear ketone carbonyl signal in the APT64‐NMR spectrum at 191.40 ppm (Figure S6) and the formation of **bi[13]** was supported by HRMS (Figures S25, S26). The three‐dimensional structure of the **bi[13]** compound was determined by X‐ray diffraction (XRD). Yellow needle‐shaped single crystals were obtained by slow diffusion of ethanol into a CH_2_Cl_2_ solution at room temperature. The crystallographic data for optically pure **bi[13]** are reported in Table S1 and the asymmetric unit observed in the crystal structure is shown in Figure 2a. The structure shown here is of a dimer of two [13]helicenes with (*P*)‐helicity. To the best of our knowledge, this is the first reported helicene featuring a four‐layered π‐system. The **bi[13]** helix is ∼1.2 nm long and the connecting C–C single bond bridging the two [13]helicene units is 1.625(7) Å long, which is appreciably longer than the common $C_{{\mathrm{sp}}^{3}}-C_{{\mathrm{sp}}^{3}}$ bond (1.54 Å).^[^ ^38^ ^]^ This elongated bond could be a contributing factor to the instability of **bi[13]** when subjected to any reaction conditions, as the elongation weakens the bond, making it more susceptible to breaking.

The first evidence of the formation of **[27]helicenoid** was the HRMS analysis (Figures S23, S24). The further ^1^H NMR spectrum analysis of **[27]helicenoid** revealed 26 aromatic protons, two different methoxy signals and eight *t*‐butyl signals, proving the unsymmetrical structure of **[27]helicenoid** (Figure S5). Crystals of **[27]helicenoid** were grown by slow evaporation of a CH_2_Cl_2_ and ethanol solution of racemic **[27]helicenoid** to give yellow needles of few microns in size. However, these crystals were unsuitable for single‐crystal XRD analysis. Instead, they proved to be well suited for electron diffraction (ED) measurements (Table S1).^[^ ^39^ ^]^

**Figure 3:**
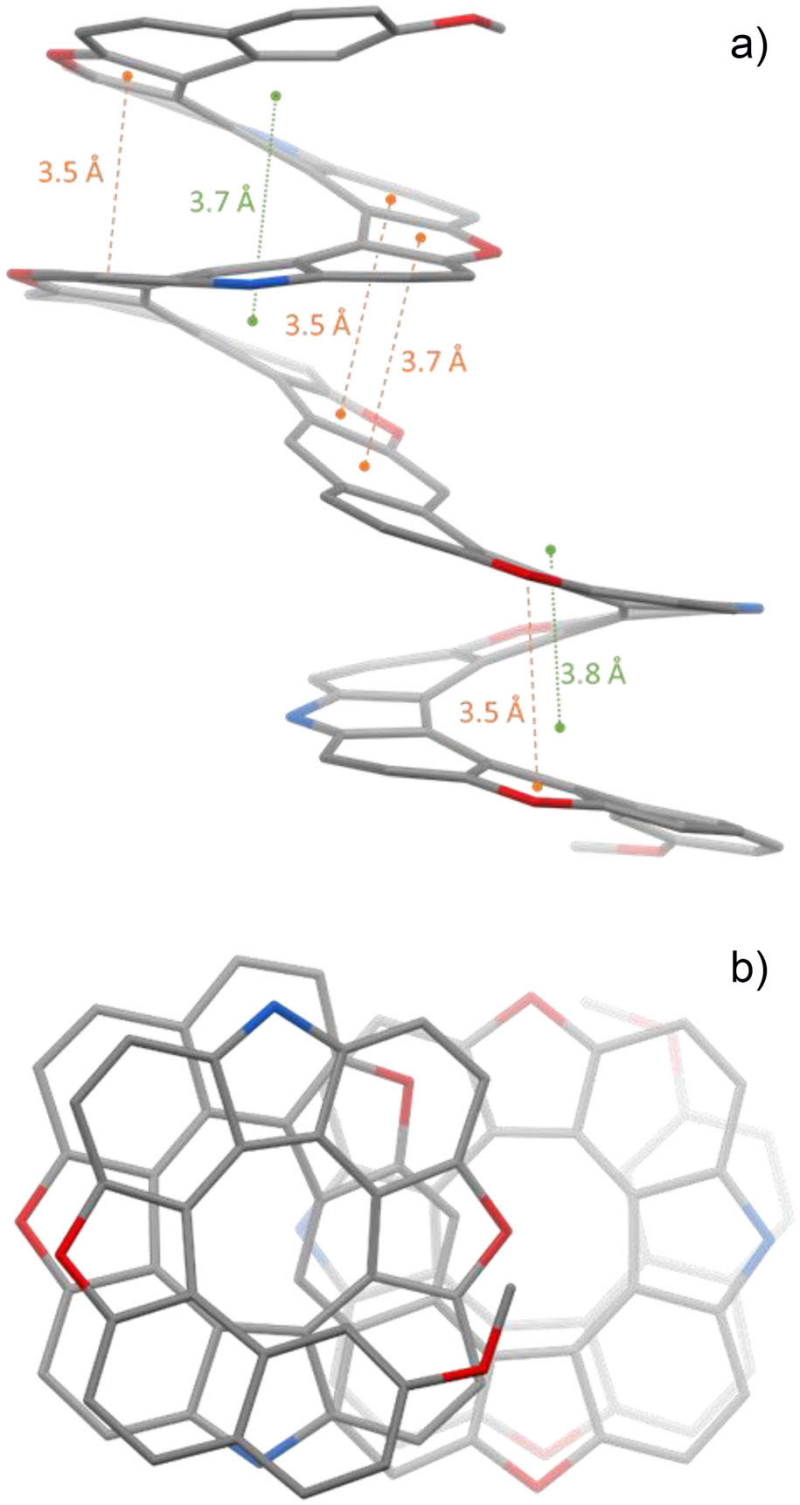
a) Simplified molecular structure of (*PM*)‐**[27]helicenoid**. The average distances between consecutive turns are reported in green, while the closest stacking distances, involving furan rings, are shown in orange. Color code: C = grey, O = red, N = blue. Alkyl groups and hydrogen atoms are omitted for clarity. See Supporting Information for the distances measurement method. b) The same molecule viewed along the helix axis, showing the staggered helices with opposite chirality.

The compound shows some polymorphism (see Supporting Information for more details). The crystal structure was solved in the orthorhombic space group *Pna*2_1_ which exhibits two independent molecules per asymmetric unit (Figure S1). The **[27]helicenoid** features both the ( *m* ) and ( *p* )‐enantiomer of the [13]helicene precursor, and both ( *mp* ) and ( *pm* )‐enantiomers of the **[27]helicenoid** are present in the crystal structure, for a total of 8 molecules in the unit cell. The crystal packing does not feature intermolecular π···π interactions and is rather loose, leaving ca. 12% of void volume in the unit cell.^[^ ^39^ ^]^ The solid‐state structure of the **[27]helicenoid** is the first example of a four‐layered fully conjugated heterohelicene (Figure 3). The helical pitch is around 3.6–3.7 Å (Figure S2), that is, slightly looser than for the [13]helicene precursor (3.5 Å, see Supporting Information). The ring‐by‐ring inter‐planar distances vary smoothly along the helix and are minimal between furan rings (3.4–3.5 Å, Figure S3), which points to favorable interlayer π···π interactions,^[^ ^40^ ^]^ regardless of a highly strained structure.

**Figure 4:**
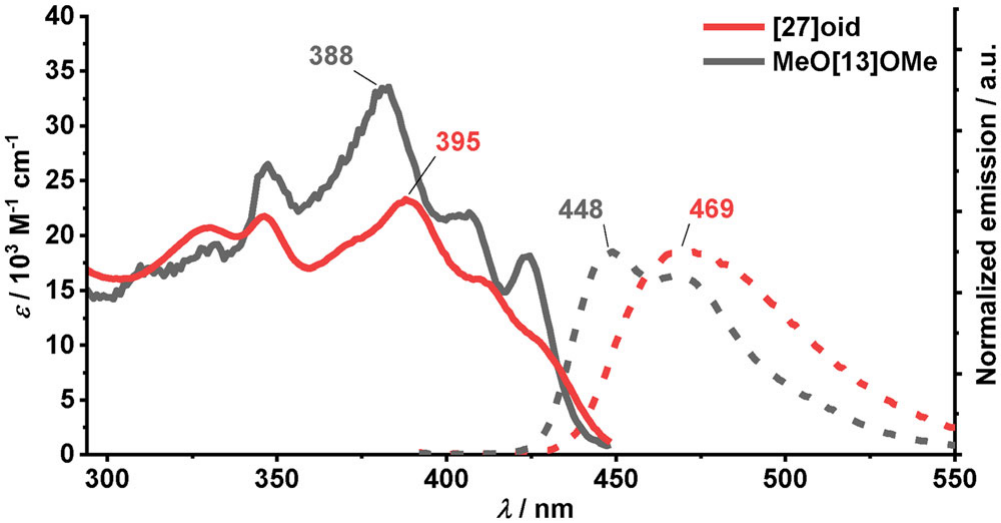
Absorption and emission spectra of **[27]helicenoid** and **MeO[13]OMe** recorded in CH_2_Cl_2_. Absorption spectra are represented by a solid line and emission by a dashed line.

**Absorption and emission properties**. The UV–vis absorption and fluorescence spectra of **[27]helicenoid** were measured in CH_2_Cl_2_ at room temperature and compared to previously reported spectra of **MeO[13]OMe** (Figures 4 and S9, S10). **[27]Helicenoid** shows reduced molar absorption coefficient by 30% compared to the [13]helicene (*ε*_max_ ≈ 33 500 for **MeO[13]OMe** versus *ε*_max_ ≈ 23 000 m ^−1^cm^−1^ for **[27]helicenoid**). When comparing the absorption spectra of the **MeO[13]OMe** and **[27]helicenoid**, the vibronic progression of the two are strikingly similar. The key difference in the absorption spectra is a small redshift of 460 cm^−1^ of the strongest electronic transition in the visible spectrum, going from **MeO[13]OMe** to **[27]helicenoid**. The absorption band edge is almost identical for the two species. The remarkable observation that the absorption spectrum remains almost unaffected after doubling the number of aromatic units in the system, suggests that the effective conjugation length (ECL) in this system has reached its limit. This can also be seen by inspecting the natural transition orbitals (NTOs, Figure S16) as the first several transitions are localized in one [13]helicene unit, extending only to the naphthol part of the second [13]helicene. When comparing the emission spectra of these two species, a small redshift of 1000 cm^−1^ is observed. Upon dimerization, the fluorescence quantum yield, *ϕ*_fl_ has been almost halved (0.18 for **[27]helicenoid** versus 0.28 for **MeO[13]OMe**), indicating new non‐radiative decay paths introduced in the annulated dimer. The inspection of the natural transition orbitals (NTOs, Figure S16) of the first two electronic transitions in **[27]helicenoid** shows that both the S_0_→S_1_ and S_0_→S_2_ are localized in the [13]helicene unit, extending only to the naphthol part of the second [13]helicene. This could be the reason why the absorption spectrum changes only slightly for the **[27]helicenoid**.

**Figure 5:**
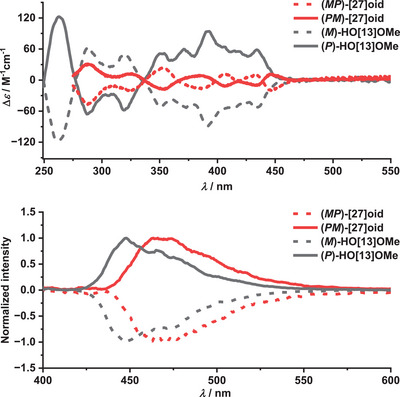
ECD (top) and CPL (bottom) spectra of **HO[13]OMe** (black) and **[27]helicenoid** (red) in CH_2_Cl_2_ at room temperature.

**Chiroptical properties**. The two enantiomers of **[27]helicenoid** were successfully resolved by HPLC on a chiral stationary phase (for details, see Supporting Information, Figure S8) to investigate its chiroptical properties and compare them to the starting material **HO[13]OMe**. As shown in Figure 5 (top), the ECD spectra of the first and second fraction recorded in CH_2_Cl_2_ revealed perfect mirrorimage relationships. The first HPLC‐eluted enantiomer displayed the positive‐to‐negative Cotton effect.

The mirror image peaks were observed for the second eluting enantiomer. Its absolute configuration was assigned by comparison of the experimental ECD with the TDDFT‐simulated ECD spectra (Figure S15). Thus, the first and second fractions correspond to (*pm* )‐ and (*mp* )‐enantiomers, respectively. The strongest ECD band of **[27]helicenoid** (*Δε* ∼ 40 m ^−1^cm^−1^) is observed in the UV region while the strongest band (*Δε* ∼ 20 m ^−1^cm^−1^) in the visible part of the spectrum is located just above 350 nm. This is a significant decrease going from the **HO[13]OMe**. The ECD spectra of both enantiomers of the **[27]helicenoid** exhibit alternating positive and negative Cotton effects above 330 nm, in contrast to **HO[13]OMe**, where the bands above this threshold are uniformly positive or negative. This pattern likely results from the combination of (*p* )‐ and ( *m* )‐helicity in the **[27]helicenoid**, contributing to opposite Cotton effects and potentially diminishing the absorbance of circularly polarized light. The enantiomers of **[27]helicenoid** displayed mirror‐imaged CPL spectra in CH_2_Cl_2_ (Figure 5, bottom and Figures S11, S12). The *g*_lum_ value of 5.5 × 10^−3^ was recorded for **[27]helicenoid** at 469 nm and for **HO[13]OMe** the *g*_lum_ was 4.2 × 10^−3^ at 448 nm, which is in the same order of magnitude observed for other aza‐ and heterohelicenes.^[^ ^28^, ^33^ ^]^

To discern whether this difference in chiroptical properties is unique to the fusing of two moieties of opposite helicity and the “telephone cord” structural motif, or simply a result of the transition energies shifting due to further πsystem extension, we modeled a simplified structure of **[27]helicenoid** with density functional theory. Time‐dependent density functional theory (TD‐DFT), capable of treating excited electronic states, has become an established approach for modelling the spectroscopy of helicenes.^[^ ^41^, ^42^, ^43^, ^44^ ^]^

In this simplified structure, we replace the bulky *t*‐butyl and propyl groups with methyl groups. For comparison, we also model the **HO[13]OMe** structure with and without the bridging (furan) structure, as this bridge itself already extends the original **HO[13]OMe** moiety. A detailed discussion of the computational results can be found in the Supporting Information (Figures S13–S16 and Tables S2, S3).

**Figure 6:**
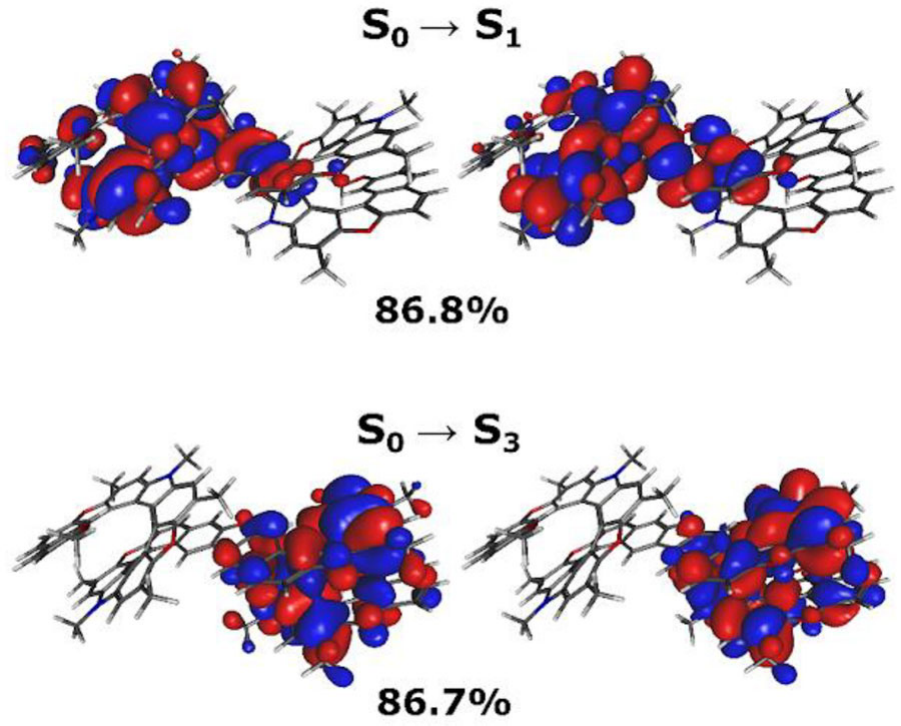
NTOs of the first two electronic singlet transitions in the simplified **[27]helicenoid** structure and their occupation numbers. 90% of electronic density shown.

Both the “telephone cord” structure and the system elongation contribute to the complex structure of the ECD spectrum of **[27]helicenoid**. First, the **HO[13]OMe** introduces a set of excited states with opposite optical rotations. The addition of a furan bridge extends the π‐system, lowering the excitation energies of transitions localized on the **HO[13]OMe** moiety of the **[27]helicenoid** which is better coupled to the furan bridge. This is possible as the furan bridge is not connected symmetrically and will therefore favor delocalization to one of its sides. This stabilization is highest for the first transition, where it amounts to −0.10 eV. This can also be seen from the NTOs (Figure S16), where electronic density is shifted into the bridging area, though does not cross over it, signaling a break in the π‐system. From the NTOs of the **[27]helicenoid** depicted in Figure 6, we observe that the lowest energy electronic transitions, stay localized on either half of the **[27]helicenoid**. The bridging area is involved in the transition, though it does not cross over it, signaling a break in the π‐system.

When both ( *m* )‐ and ( *p* )‐moieties are bridged together, the symmetry is broken and we see two opposite and interlaced patterns of states, separated by the previously mentioned stabilization energy. The result is a strongly oscillating ECD spectrum, at least in the lower energy (and lower density of states) region.

## Conclusion

In this work we described the synthesis of the first four‐layered [27]helicenoid. Starting from a racemic mixture of the previously described [13]helicene, we expected to obtain the [27]helicene via a copper catalyzed dimerization/cyclization reaction of the [13]helicene. Surprisingly, this reaction did not give the targeted [27]helicene by a C–C coupling in 1,1′ position, but instead led to the formation of an analogue helicenoid, formed by C–C coupling in the 1,3′ position. The resulting **[27]helicenoid** displays the same molecular mass as the **[27]**, but is a heterochiral dimer of two [13]helicenes (i.e., the ( *m* )‐ and ( *p* )‐enantiomers). The structural features and the (chir)optical properties of the **[27]helicenoid** were determined by electron diffraction, UV‐vis spectroscopy, and ECD and CPL measurements. For the latter analyses, the two enantiomers of the racemic **[27]helicenoid** were successfully separated. Supported by TD‐DFT studies, it was observed that the **[27]helicenoid** is able to rotate circularly polarized light of both ( *m* )‐ and (*p* )‐chirality. This observation could also explain why the UV‐vis spectra of the [13]helicene and the **[27]helicenoid** look strikingly similar. Both structures feature a four‐layered π‐system with four helical turns, which is the longest reported helicene‐like system.

## Supporting Information

All synthetic and experimental details as well as computational details. The authors have cited additional references within the Supporting Information.^[^ ^45^, ^46^, ^47^, ^48^, ^49^, ^50^, ^51^, ^52^, ^53^, ^54^, ^55^, ^56^, ^57^, ^58^, ^59^, ^60^, ^61^, ^62^, ^63^, ^64^ ^]^ Crystallographic data and atomic coordinates for the structures reported in this paper have been deposited with the Cambridge Crystallographic Data Centre (deposition numbers CCDC 2410748, 2410749, 2421786). These data can be obtained free of charge from The Cambridge Crystallographic Data Centre via www.ccdc.cam.ac.uk/structures.

## Conflict of Interests

The authors declare no conflict of interest.

## Supporting information



Supporting Information

## Data Availability

The data that support the findings of this study are available in the Supporting Information of this article.
