# Supplementary material for: Helicenes with Four Helical Turns: Dimerization of [13]Helicenes to [27]Helicenoids
Source: Angew Chem Int Ed Engl. 2025 May 27;64(29):e202506328. doi: 10.1002/anie.202506328 (PMC12258660; doi:10.1002/anie.202506328)
Supplement: Supplementary file 1 — Supporting Information [file ANIE-64-e202506328-s001.docx]

**Helicenes with four helical turns: Dimerization of [13]helicenes to [27]Helicenoids**

Matea Sršen,^[a]^ Stephan K. Pedersen, Tomislav Rožić, Arianna Lanza, Michael Pittelkow^[a]^

[a] University of Copenhagen, Department of Chemistry Universitetsparken 5, DK-2100 Copenhagen Ø, Denmark.

E-mail: pittel@chem.ku.dk

**Supporting Information**

[1. General Experimental Procedures 2](#_Toc192524917)

[2. Crystallographic Data 4](#_Toc192524918)

[3. Synthetic Procedures 10](#_Toc192524919)

[3.1. Synthesis of compound **bi[13]** 10](#_Toc192524920)

[3.2. Synthesis of compound **[27]helicenoid** 10](#_Toc192524921)

[4. HPLC Separations 14](#_Toc192524922)

[5. Optical Properties 15](#_Toc192524923)

[5.1 Circularly Polarized Luminescence 16](#_Toc192524924)

[6. Computational Details 17](#_Toc192524925)

[7. NMR Spectra 23](#_Toc192524926)

[8. HRMS Spectra 26](#_Toc192524927)

[9. References 30](#_Toc192524928)

# General Experimental Procedures

Unless otherwise stated, commercially available materials were used without further purification. All solvents used were purchased in HPLC grade from VWR and used without further purification. Abs. EtOH and petroleum spirit (bp: 40–65 °C) used was technical grade. Anhydrous CH_2_Cl_2_ was obtained by drying over molecular sieves (4 Å) for at least 24 h prior to use. Flash column chromatography was performed using silica gel (particle size: 40-63 μm) and Celpure celite 545. Recycling preparative gel permeation chromatography (GPC) was performed with a LaboACE LC-5060 (Japan Analytical Industry) equipped with polystyrene gel columns (JAIGEL-2HR-40, Japan Analytical Industry) using CH_2_Cl_2_ or CHCl_3_ as eluent. TLC plates used are Merck‑DC‑Alufolien SiO_2_ 60 F254 0.2 mm thick.

**NMR spectroscopy** was performed on a BRUKER Avance III 500 Ultrashield Plus with a 5 mm CPDCH CryoProbe with enhanced ^13^C sensitivity at 500 MHz and 126 MHz for ^1^H and ^13^C NMR respectively. All ^1^H-NMR and ^13^C‑NMR spectra are referenced to the residual solvent peak; (CD_2_Cl_2_ δ_H_ = 5.32 ppm and δ_C_ = 53.84 ppm). Chemical shifts are referenced to the ppm scale and coupling constants are expressed in Hertz (Hz). Apparent multiplicities are reported as s (singlet), d (doublet), t (triplet), q (quartet), dd (doublet of doublets) or m (multiplet). NMR data were processed using MestReNova v. 14.3.1-31739.

**UV-vis** was measured on a Cary 100 Bio UV-visible spectrophotometer. All UV-vis measurements were performed in CH_2_Cl_2_ at 20 °C in a 1.0 cm quartz cuvette.

**Fluorescence spectra** were measured on a Perkin Elmer Luminescence Spectrometer LS 50 B with a double monochromator for both detection and excitation. The fluorescence measurements were conducted at 20 °C in CH_2_Cl_2_ on a sample with absorbance below 0.1 of the highest *λ*_max_ and recorded in a 10 mm quartz cuvette.

All the **calculations** reported in this chapter were obtained with the ORCA 5.0.4^[1]^ software package and its default resolution-of-identity and chain of spheres (RIJCOSX) options for density functional theory (DFT). We applied the range-separated CAM-B3LYP functional in conjunction with the D3BJ empirical dispersion correction and the def2-TZVP basis set. Solvation was treated implicitly with CPCM for dichloromethane.

The full spectrum (up to around 325 nm) was only accessible using the Tam-Damcoff approximation (TDA-DFT), so the locations of the first four peaks were confirmed by computing 30 singlet excitations with full TD-DFT. ORCA results were analyzed using the Multiwfn software^[2]^ package. Natural transition orbitals (NTOs) were computed using ORCA and visualized in Gabedit.^[3]^ The spectrum was plotted using the default length gauge result, which matched both those of the velocity and length gauge in the smaller, benchmark TD-DFT result. See Section 6 for details.

For **chiral HPLC**, 5-AmyCoat column 4.6 × 250 mm equipped with two UFLC Shimadzu LC-20AD HPLC pumps was used. UV-detector wavelengths were 254 and 354 nm. A semipreparative chiral COSMOSIL Cholester column (250 × 10 mm i.d) with eluent system of methanol/CH2Cl2; flow rate: 2 mL/min; detected by absorption at 450 nm was used on GPC.

**Electronic Circular dichroism (ECD) spectroscopy** analyses were performed on a Jasco J-1500 CD Spectrometer. CD experiments were carried out using a square 10 mm quartz cuvette. Scans were performed at 25 °C over a wavelength range of 225–600 nm with bandwidth of 2.0 nm, scanning speed of 200 nm/min, digital integration time of 0.125  or 1 s, data pitch of 0.1 nm, and CD scale of 200 mdeg. The CD spectra are an average of 3 scans. The collected data was processed in OriginPro 2023. Background spectrum of CH_2_Cl_2_ was subtracted from the collected data.

**Circularly polarized luminescence (CPL) spectroscopy** analyses were performed on a Jasco CPL‑300 CPL spectrometer. Experiments were carried out using a square 10 mm quartz cuvette at room temperature with HPLC-grade dichloromethane as solvent. Data pitch was either 0.5 or 1.0 nm, CD scale was 10000 mdeg with a digital integration time of 16 s, excitation and emission bandwidth were 20.00 nm and 10.00 nm respectively, scanning speed was 20 nm/min, and the spectra were averaged over 9 scans. Detector bias voltage of either 650 V or 700 V was used. The collected data were processed in OriginPro 2023.

All ECD and CPL measurement were performed in the same cuvette.

**MALDI-TOF‑HRMS and ESI-HRMS** was performed on a SolariX ESI/MALDI FTMS spectrometer, with dithranol as matrix, by Anette Andersen. External calibration of the spectrometer was conducted with sodium trifluoroacetate cluster ions.

**Crystal data and structure refinement** statistics are given in Table S1. Crystallographic data and atomic coordinates for the structures reported in this paper have been deposited with the Cambridge Crystallographic Data Centre (deposition numbers CCDC 2410748, 2410749, 2421786). These data can be obtained free of charge from The Cambridge Crystallographic Data Centre via www.ccdc.cam.ac.uk/structures.

# Crystallographic Data

- 1. **Crystal structure of bi[13]**

Crystals of **bi[13]** were grown by slow evaporation from ethanol and dichloromethane and the structure was determined with single-crystal x-ray diffraction (SCXRD). The SCXRD data were collected at 100 K on a Bruker D8 VENTURE diffractometer with a mirror-optics monochromated microfocus X-ray source (MoKα radiation, λ = 0.71073 Å), a Bruker PHOTON 100 CMOS detector and an Oxford Cryosystems nitrogen Cryostream 800. All data were integrated with SAINT V8.40B^[4]^ and a multi-scan absorption correction was applied using SADABS 2016/2.^[5]^ The structures were solved by dual methods with SHELXT^[6]^ and refined by full-matrix least-squares methods against *F*^2^ using SHELXL^[7]^, via the interfaces SHELXLe^[8]^ and Olex2^[9]^. All non-hydrogen atoms were refined with anisotropic displacement parameters. H-atoms were placed on calculated positions and refined using a riding model with their U_iso_ values constrained to 1.5 times the U_eq_ of their pivot atoms for terminal sp^3^ carbon atoms and 1.2 times for all other carbon atoms. Disordered moieties were refined using bond lengths restraints and displacement parameter restraints. Crystal data and structure refinement statistics are given in Table S1.

- 1. **Crystal Structure of [27]helicenoid**

Crystals of **[27]helicenoid** could only be grown to a size of few microns, and the structure was determined with 3D electron diffraction (3D ED). To this end, a drop of the suspension containing crystals was deposited on a glass slide and let to dry in the air. A 300-mesh copper TEM grid coated with a continuous film of ultrathin amorphous carbon (from EMS) was then gently tapped on the dried powder. 3D ED experiments were performed at ambient temperature and at 170 K in high vacuum on a Rigaku XtaLAB Synergy-ED, equipped with a LaB_6_ source operating at 200 kV (λ = 0.0251 Å) and a Rigaku HyPix‑ED detector. For the low-temperature experiment, the sample grid was cooled in vacuum (ca 6 K/min) with a Gatan Elsa cryoholder. Series of diffraction patterns were collected on over 20 crystals during continuous rotation of the specimen. Selected area apertures with an apparent diameter of 1 or 2 μm were used to delimit the diffracting region of the crystals. The program CrysAlis Pro^[10]^ was used to control the data collection and multiple illumination conditions, detector distances and data collection parameters were tested to improve the data quality. Nevertheless, all crystals diffracted to a resolution of 1 Å at best, even at 170 K.

The majority of the crystals could be indexed with an orthorhombic unit cell (see Table S1), and two of the measured crystals could be indexed with a monoclinic unit cell. The space group assignment for the orthorhombic datasets was not trivial, because different datasets showed variable violations of the systematic extinctions. The variable symmetry and subtle polymorphism could be rationalized with the interplay of the helicoidal shape (i.e. approximatively cylindrical) and the absence of strongly directional intermolecular interactions (e.g. H–bonds). In absence of steric or energetic factors directing the mutual orientation of the molecules, slight positional variations can occur (e.g. a rotation around the main axis) within the same crystal and, even more so, throughout the entire population of crystals, which in fact shows a range of possible packing symmetries. Moreover, a similar subtle effect can be due to the very flexible side groups, whose conformational variations could break the symmetry of an otherwise more ordered packing of the rigid helicoidal cores.

A dataset with high completeness and recognizable *Pna*2_1_ symmetry allowed for structure solution of the orthorhombic phase. The crystal structure was solved ab initio with SHELXD^[11]^ using Olex2^[9]^. The least-squares refinement in kinematic approximation was carried out with SHELXL^[7]^, via the interfaces SHELXLe^[8]^ and Olex2^[9]^, using the scattering factors for electrons^[12]^. The positions of all atoms of the [27]helicenoid core were refined freely. The *t*Bu and Pr side groups are slightly disordered and soft restraints were applied to maintain a consistent geometry and to mitigate for the low data/parameter ratio (the number of data is limited by the low resolution). An extinction parameter was refined to mitigate the effects of multiple diffraction. Anisotropic displacement parameters were refined for non-hydrogen atoms, while H-atoms were placed in geometrically idealized position and refined with a riding model, using tabulated distances from neutron diffraction and isotropic thermal parameters derived from the parent atom.

A structure model could also be obtained in space group *P*2_1_ for the monoclinic polymorph, by combining the datasets of 2 crystals. Again the structure features 2 independent molecules (i.e. a pair of enantiomers) in the asymmetric unit. Despite the lower symmetry, the crystal packing is extremely similar to the orthorhombic one. For this reason, we focused our attention on the orthorhombic polymorph which is the predominant form, had better data completeness and quality and yielded a model with higher precision (see Table S1).

The structure features two independent molecules in the asymmetric unit, having almost superimposable conformation, with the exception of the side groups (Figure S1).


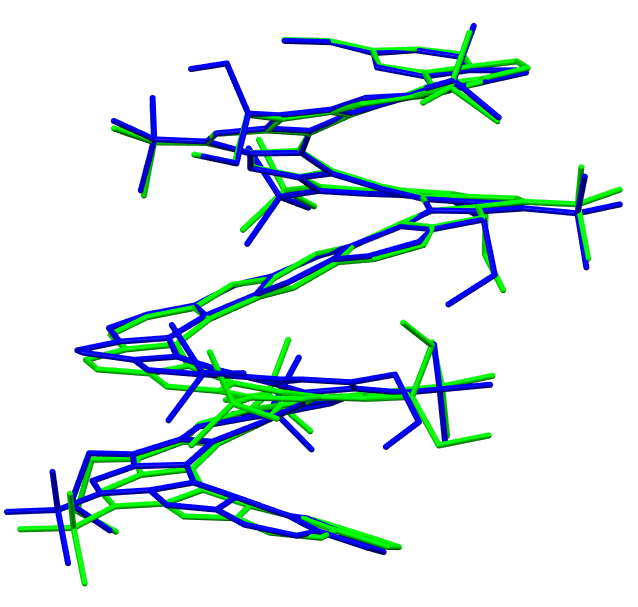


**Figure S1.** Near-superimposable conformations of the two symmetry-independent molecules in the crystal structure of **[27]helicenoid**.

Both the (*PM*) and the (*MP*) enantiomer are present in the crystal. We define the (*PM*) enantiomer as the one in which the furan oxygen in the bridge (i.e. the 14^th^ ring) points in the same direction as the right-handed (*P*) helix; the bridge is followed by a left-handed (*M*) helix. The crystal packing is rather inefficient and the structure features voids accounting for ca. 12.4% of the unit cell volume. No solvent or other guest molecules were located in the voids. The solvents used for crystallization are very volatile and, in case they were incorporated in the crystal structure, they probably can evaporate easily at ambient conditions or in the high vacuum inside the electron diffractometer.

**Table S1**. Crystal data and structure refinement for **bi[13]**·2EtOH and **[27]helicenoid**.

|  | **bi[13]·2EtOH** | **[27]helicenoid** | **[27]helicenoid (monoclinic)** |
| --- | --- | --- | --- |
| CCDC deposition number | 2410748 | 2410749 | 2421786 |
| Empirical formula | C_134_H_132_N_4_O_10_ · 2C_2_H_6_O | C_134_H_132_N_4_O_9_ | C_134_H_132_N_4_O_9_ |
| Formula weight | 2052.58 | 1942.55 | 1942.55 |
| Method | SC-XRD | 3D ED | 3D ED |
| Temperature [K] | 100 | 170 | 170 |
| Crystal system | monoclinic | orthorhombic | monoclinic |
| Space group (number) | $P2_{1}$ (4) | $Pna2_{1}$ (33) | $P2_{1}$ (4) |
| *a* [Å] | 14.1827(12) | 29.622(4) | 14.332(3) |
| *b* [Å] | 28.103(2) | 25.8426(16) | 30.810(4) |
| *c* [Å] | 14.4381(12) | 30.608(2) | 26.924(12) |
| α [°] | 90 | 90 | 90 |
| β [°] | 103.739(3) | 90 | 93.94(2) |
| γ [°] | 90 | 90 | 90 |
| Volume [Å^3^] | 5589.9(8) | 23430(4) | 11861(6) |
| *Z* | 2 | 8 | 4 |
| *ρ*_calc_ [gcm^−3^] | 1.219 | 1.101 | 1.088 |
| *μ* [mm^−1^] | 0.077 | n.a. | n.a. |
| *F*(000) | 2196 | 3461 | 1730 |
| Crystal size [mm^3^] | 0.15×0.204×0.308 | 0.001×0.002×n.a. | n.a. |
| Radiation | Mo*K_α_* (λ=0.71073 Å) | electron (λ=0.0251 Å) | electron (λ=0.0251 Å) |
| 2θ range [°] | 4.61 to 52.75 (0.80 Å) | 0.09 to 1.44 (1.00 Å) | 0.07 to 1.33 (1.08 Å) |
| Index ranges | −17 ≤ h ≤ 17 −35 ≤ k ≤ 28 −18 ≤ l ≤ 18 | −28 ≤ h ≤ 28 −25 ≤ k ≤ 25 −30 ≤ l ≤ 30 | −13 ≤ h ≤ 13 −28 ≤ k ≤ 28 −24 ≤ l ≤ 24 |
| Reflections collected | 75415 | 50325 | 24715 |
| Independent refl. (*R*_int_ / *R*_sigma_) | 20946 (0.080 / 0.068) | 21003 (0.193 / 0.258) | 14751 (0.246 / 0.386) |
| Completeness | 99.8 % | 84.6 % | 75.7 % |
| Data / Restraints / Parameters | 20946 / 110 / 1448 | 21003 / 6373 / 2747 | 14751 / 11087 / 2648 |
| Absorption correction method | empirical | multi-scan | multi-scan |
| Goodness-of-fit on *F*^2^ | 1.045 | 1.005 | 1.000 |
| Final *R* indexes  [*I*≥2σ(*I*)] | *R*_1_ = 0.0689 w*R*_2_ = 0.1679 | *R*_1_ = 0.1336 w*R*_2_ = 0.3067 | *R*_1_ = 0.1675 w*R*_2_ = 0.3681 |
| Final *R* indexes  [all data] | *R*_1_ = 0.0885 w*R*_2_ = 0.1797 | *R*_1_ = 0.1934 w*R*_2_ = 0.3542 | *R*_1_ = 0.2753 w*R*_2_ = 0.4570 |
| Max/min residuals | 0.55/−0.32 [eÅ^−3^] | 0.14/−0.13 [Å^−2^] | 0.12/−0.13 |
| Extinction coefficient |  | 13.8(19) | 25(6) |

**Analysis of the molecular geometry**

In order to measure and compare the molecular geometries, we have simplified the structures of **bi[13]**, **[27]helicenoid** and **[13]helicene** as follows, starting the from the atomic coordinates in the crystal structures or in the DFT model. All hydrogen atoms, propyl and *t*-butyl groups were removed. Subsequently, the centroids of every ring were calculated with the program Mercury^[13]^ (Figure S2A,C). The centroids were labeled to reflect their position in the sequence of condensed rings. For the asymmetric molecules of **[27]helicenoid**, the order of the rings needs to be specified. For the (*PM*) enantiomer, ring 1 is chosen as the terminal ring on the *P* helix of the molecule and ring 27 is the terminal ring at the *M* side of the molecule. The coordinates of the centroids form a simple helix which was used to determine the average intramolecular distances. The distances between each ring and the nearest one stacked above and/or below were measured in Mercury as the distances between the centroids. It takes 8 rings to form a complete turn of the helix, so ring 1 is stacked with ring 9, 2 with 10 etc. (Figure S2B). The sequence of inter-ring distances for the three examined structures can be plotted and compared visually (Figure S3). For **[27]helicenoid**, the distances were measured for both molecules in the asymmetric unit, in both the orthorhombic and the monoclinic polymorph and all show a very similar trend. Finally, to estimate the pitch of the helices, we have calculated the “centroid of each turn”, by calculating the centroid between the 1^st^ and 5^th^ ring of each turn (Figure S2B,D). This very simplified representation of the “centroid of each turn” was chosen over other options, because it enables a consistent comparison among all structures, unbiased by the presence of one incomplete turn in **[27]helicenoid**.


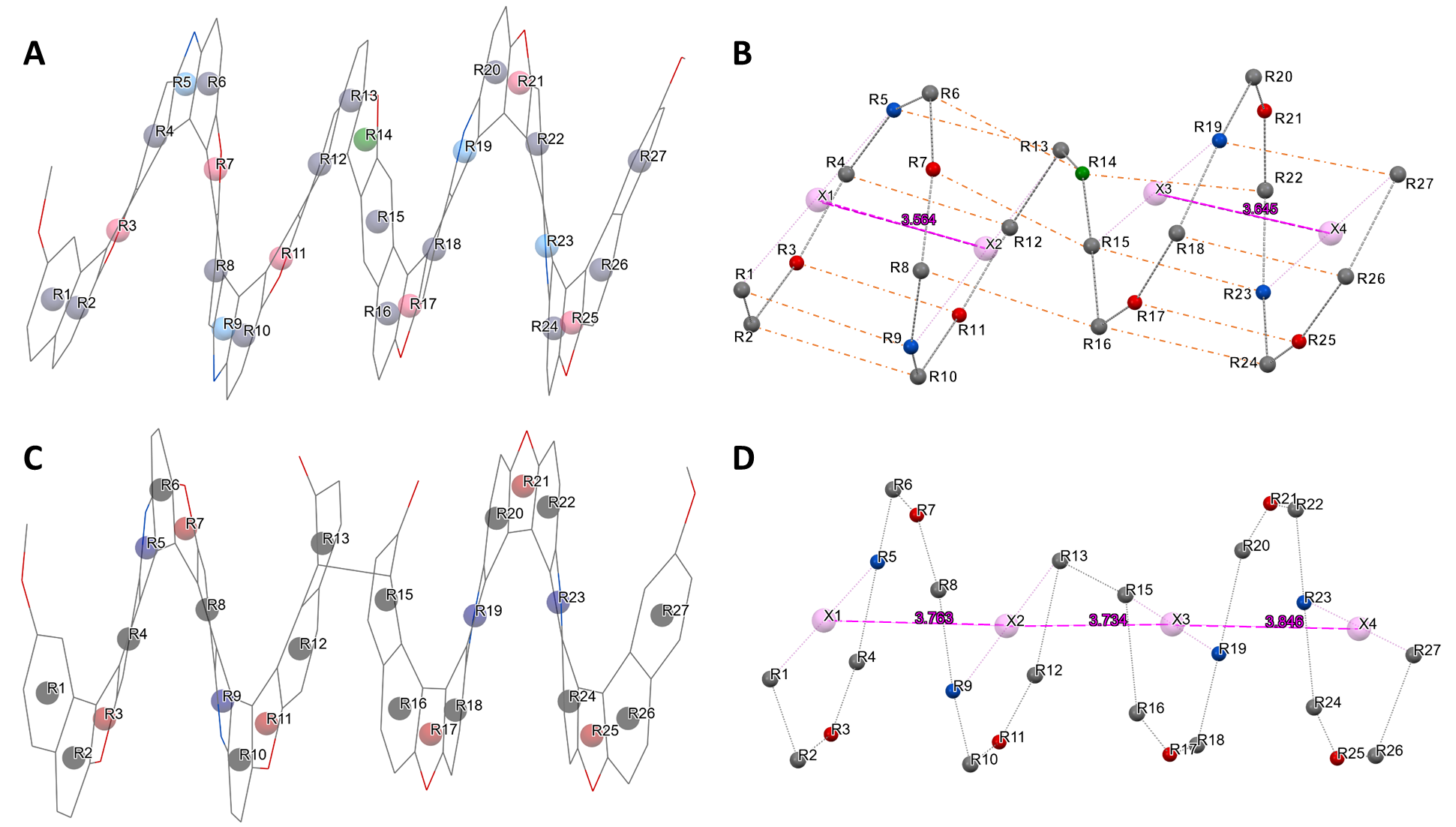


**Figure S2.** Simplified helices for **[27]helicenoid** (A,B) and **bi[13]** (C,D). For every structure model, the aliphatic chains and H atoms were removed and the centroid was calculated for every ring, and labeled R1…R27 (panel A and C). The centroids were then used to calculate the geometrical descriptors (panel B and D). The orange dashed-dotted lines show the stacking of neighboring rings. Finally, the centroids of the turns (X1…X4, pink spheres) are calculated as the midpoint of the pairs of opposite rings joined by pink dotted lines (X1=R1—R5, X2=R9—R13, X3=R15—R19, X4=R23—R27). The pitch of the helices is then estimated from the distances between consecutive X points (magenta dashed lines).


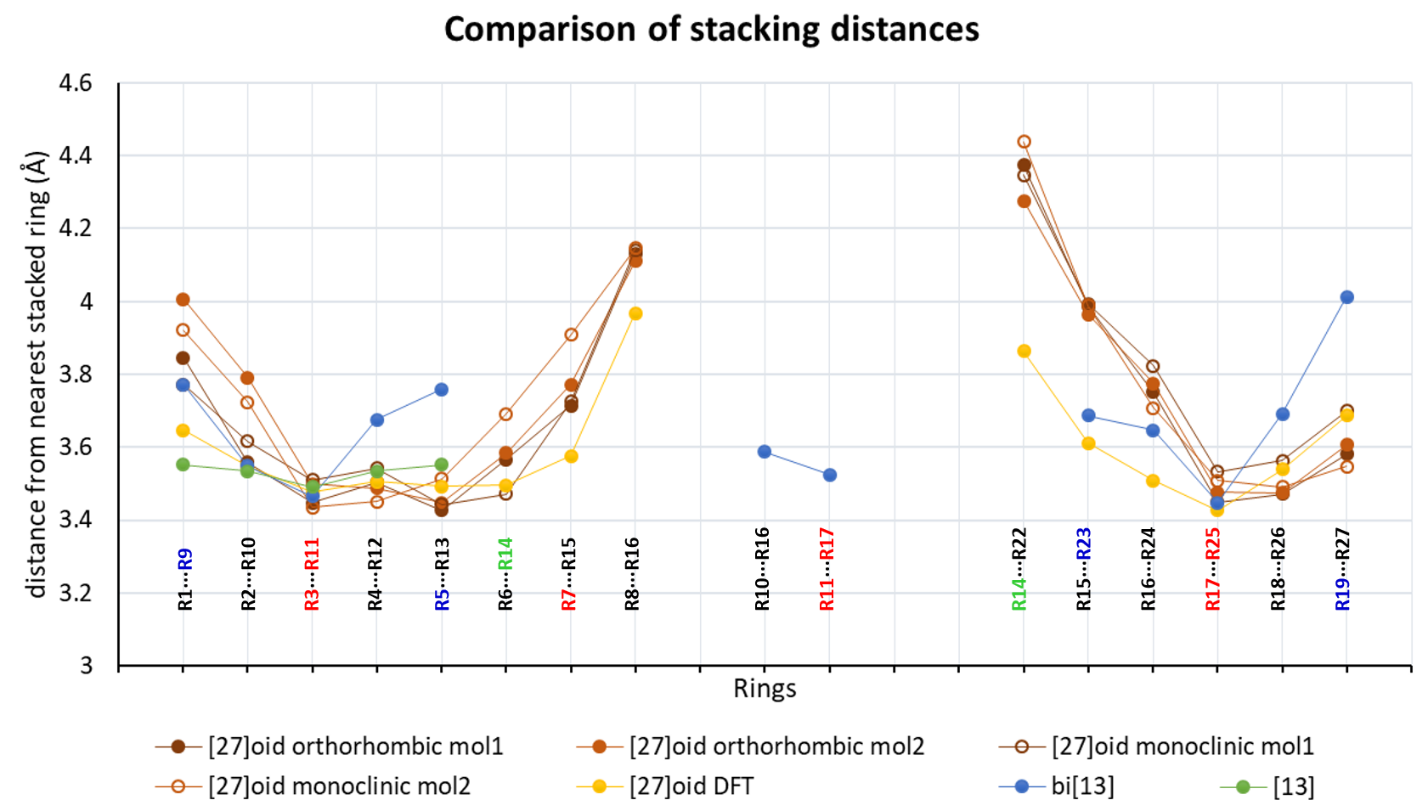


**Figure S3.** The distances between pairs of stacked rings are plotted in sequence, as they occur along the molecular skeleton, for **[13]**, **bi[13]** and **[27]helicenoid**. The rings are numbered from R1 to R27 according to Figure S2. The general stacking rule is R(n)···R(n+8), with the exception of the central part of **bi[13]**. The ring labels are color coded, to highlight the rings containing heteroatoms: blue = pyrrole, red = furan, green = central furan bridge. All distances are determined from the crystal structures, with the exception of the series “**[27]helicenoid** DFT”. For **[27]helicenoid**, the measurements of both symmetry-independent molecules in both polymorphs are reported, showing a consistent trend. The measurements for the precursor **[13]** is taken from literature^[14]^ (CSD refcode: WOYJEC).

# Synthetic Procedures

Compound **HO[13]OMe** was prepared according to literature procedure.^[14]^

- 1. Synthesis of compound **bi[13]**

**Procedure:** To a round-bottomed flask equipped with a magnetic stir bar was added (*P*)-**HO[13]OMe** (50 mg, 0.051 mmol, 1.0 eq.) and dissolved in dichloromethane (0.51 mL). To the stirred reaction was added Cu^II^Cl(OH)-TMEDA (2.4 mg, 5.1 μmol, 0.1 eq.), the reaction was fitted with an oxygen atmosphere and stirred overnight. To the completed reaction was added water (5 mL) and the mixture was extracted with dichloromethane (3 × 5 mL), dried with MgSO_4_, filtered and the solvents removed under reduced pressure.

**NMR Characterization:** see discussion in the manuscript and Figure S4 below.

**HR-MS (MALDI):** calcd. for C_134_H_134_N_4_NaO_10_ [M+Na]^+^ is 1983.0025, found 1982.9650.

See also Figures S25-S26.

- 1. Synthesis of compound **[27]helicenoid**

**Procedure:** To a round-bottomed flask, equipped with a magnetic stir bar, was added **HO[13]OMe** (51 mg, 0.051 mmol) and dissolved in chlorobenzene (0.51 mL, 220 ppm H_2_O). To the stirred reaction was added Cu^II^OTf_2_ (11 mg, 0.031 mmol, 0.6 eq.), the reaction was fitted with a nitrogen atmosphere, heated to 100 °C and stirred overnight. To the completed reaction was added water (5 mL) and the mixture was extracted with dichloromethane (3 × 5 mL), dried with MgSO_4_, filtered and the volatiles removed under reduced pressure. The crude product mixture was purified with flash column chromatography (gradient of 1:40 to 1:20 ether:heptane) to afford **[27]helicenoid** as a yellow solid.

**Yield:** 58% (28 mg, 0.014 mmol).

**^1^H-NMR:** (500 MHz, CD_2_Cl_2_) δ 7.51 – 7.46 (m, 2H), 7.32 (d, *J* = 8.6 Hz, 1H), 7.29 – 7.23 (m, 2H), 7.17 – 7.12 (m, 3H), 7.12 – 7.08 (m, 2H), 7.05 (d, *J* = 8.5 Hz, 2H), 6.91 (d, *J* = 1.7 Hz, 1H), 6.86 (s, 1H), 6.84 (d, *J* = 8.6 Hz, 1H), 6.80 (s, 1H), 6.74 (s, 1H), 6.67 (d, *J* = 2.5 Hz, 1H), 6.45 (dd, *J* = 8.6, 2.5 Hz, 1H), 6.23 (s, 1H), 6.10 – 6.06 (m, 2H), 6.04 (d, *J* = 2.5 Hz, 1H), 5.98 (s, 1H), 5.91 (d, *J* = 8.7 Hz, 1H), 5.85 (s, 1H), 4.24 – 4.19 (m, 2H), 4.13 – 4.08 (m, 2H), 3.87 – 3.80 (m, 2H), 3.09 – 2.91 (m, 2H), 2.57 (s, 3H), 2.36 – 2.26 (m, 2H), 2.07 – 2.03 (m, 2H), 1.95 (s, 9H), 1.93 (s, 3H), 1.85 (s, 9H), 1.76 (s, 9H), 1.71 (s, 9H), 1.49 (s, 9H), 1.31 – 1.28 (m, 4H), 1.27 (s, 9H), 1.20 (s, 9H), 1.07 (t, *J* = 7.2 Hz, 3H), 1.00 (t, *J* = 7.3 Hz, 3H), 0.88 (t, *J* = 7.1 Hz, 3H), 0.19 (s, 9H).

^1^**^3^C-NMR:** (126 MHz, CD_2_Cl_2_) δ 154.93, 154.10, 153.78, 153.36, 152.57, 152.29, 151.65, 151.43, 149.19, 149.17, 149.00, 148.85, 148.73, 147.61, 147.53, 137.38, 137.12, 137.04, 136.76, 136.68, 136.66, 136.31, 136.24, 131.58, 131.52, 131.14, 131.02, 131.00, 130.92, 130.54, 130.20, 129.40, 129.19, 128.00, 127.28, 126.62, 126.55, 126.16, 125.86, 125.63, 125.20, 125.10, 124.73, 124.31, 124.06, 124.01, 123.43, 122.92, 122.36, 121.74, 121.72, 121.69, 120.70, 120.50, 120.47, 120.39, 119.58, 119.36, 119.05, 118.54, 118.15, 117.79, 117.21, 115.46, 115.33, 115.10, 114.81, 114.58, 114.26, 114.22, 113.96, 110.82, 109.45, 109.39, 109.09, 109.02, 107.14, 105.35, 105.09, 104.58, 103.80, 103.77, 103.48, 103.30, 102.83, 102.73, 102.59, 102.39, 53.87, 53.13, 45.51, 45.15, 44.70, 44.20, 35.23, 35.06, 34.96, 34.91, 34.77, 34.71, 34.50, 33.30, 30.48, 30.44, 30.37, 30.34, 30.28, 30.13, 30.07, 29.40, 28.62, 23.26, 23.04, 22.25, 12.63, 12.22, 12.18, 11.53.

See also Figures S17-S22.

**Figure S4.** ^1^H NMR (500 MHz, CD_2_Cl_2_) spectrum of **HO[13]OMe** (top) and partially purified **bi[13]** (bottom). The color-marked peaks correspond to protons depicting the symmetrical nature of **bi[13]** diketone. Blue are singlets corresponding to tBu groups on the carbazole parts and green are the methoxy groups at the terminal naphthol units. The black asterisk corresponds to a singlet of a proton (H_a_) in the α‑position to the carbonyl group. Orange marked doublets are part of the naphthol unit where the two [13]helicene units couple.

**Figure S5.** ^1^H NMR spectrum (500 MHz, CD_2_Cl_2_) of **HO[13]OMe** (top) and **[27]helicenoid** (bottom). Marked are peaks corresponding to protons depicting the unsymmetrical nature of the **[27]helicenoid** in comparison to the [13]helicene. Blue are singlets corresponding to tBu‑groups on the carbazole parts and green are the two methoxy groups at the end of the combined (P)- and (M)-helicene(s) of the molecule.

*****

**Figure S6.** APT64-NMR (126 MHz, CD_2_Cl_2_) spectrum of partially purified **bi[13]** with black asterisk corresponding to carbonyl group of **bi[13]** diketone.

**Figure S7.** ^1^H–^13^C HSQC NMR (500 MHz, CD_2_Cl_2_) spectrum of partially purified **bi[13]** showcasing the sp^3^ hybridized C atom at 57.0 ppm and the corresponding proton at 2.63 ppm.

# HPLC Separations

Racemic **[27]helicenoid** was dissolved in heptane and the enantiomers separated using HPLC with chiral stationary phase (Kromasil 5-AmyCoat column 4.6 mm x 250 mm, particle size 3 μm, 0.7 mL/min, 0.7% isopropanol in heptane, 25 μL injection).


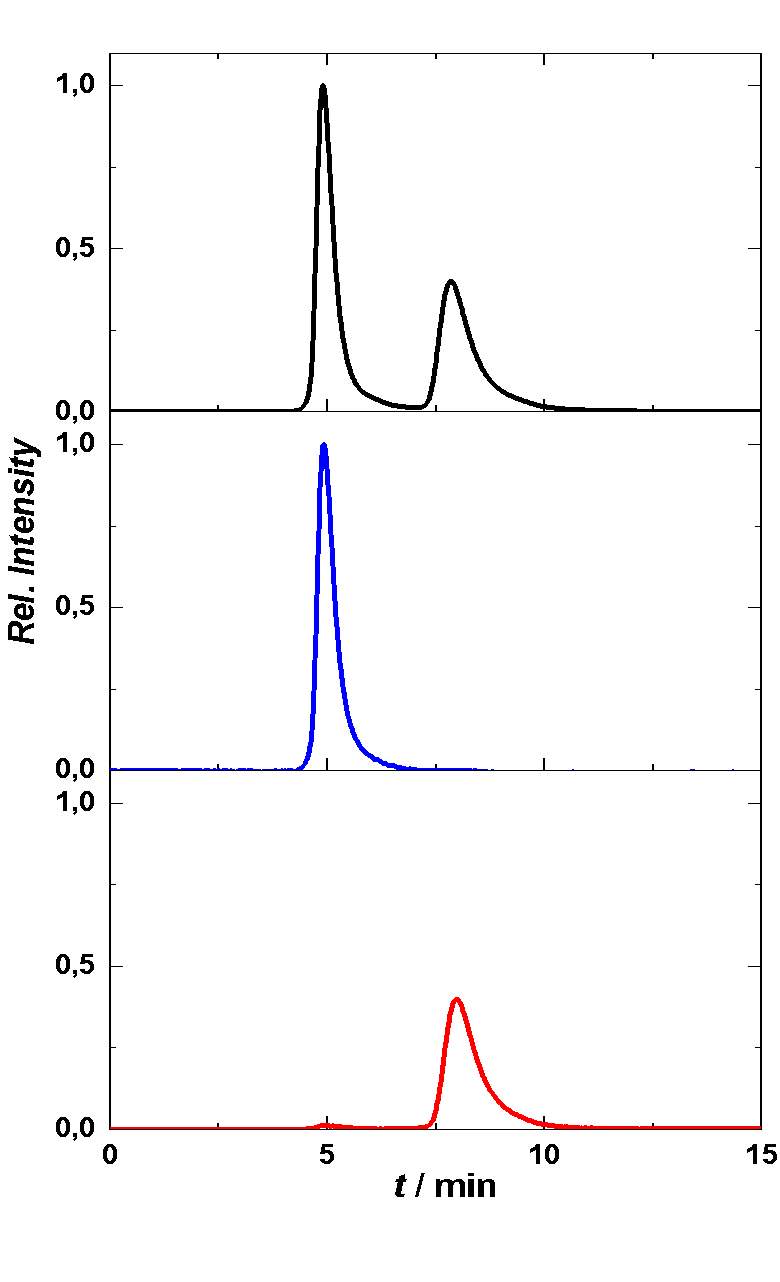


**Figure S8.** HPLC chromatogram of **[27]helicenoid** with (PM)-enantiomer (blue) eluting first followed by (MP)-enantiomer (red).

# Optical Properties

**Figure S9.** Absorption spectrum of **[27]helicenoid** in CH_2_Cl_2_.


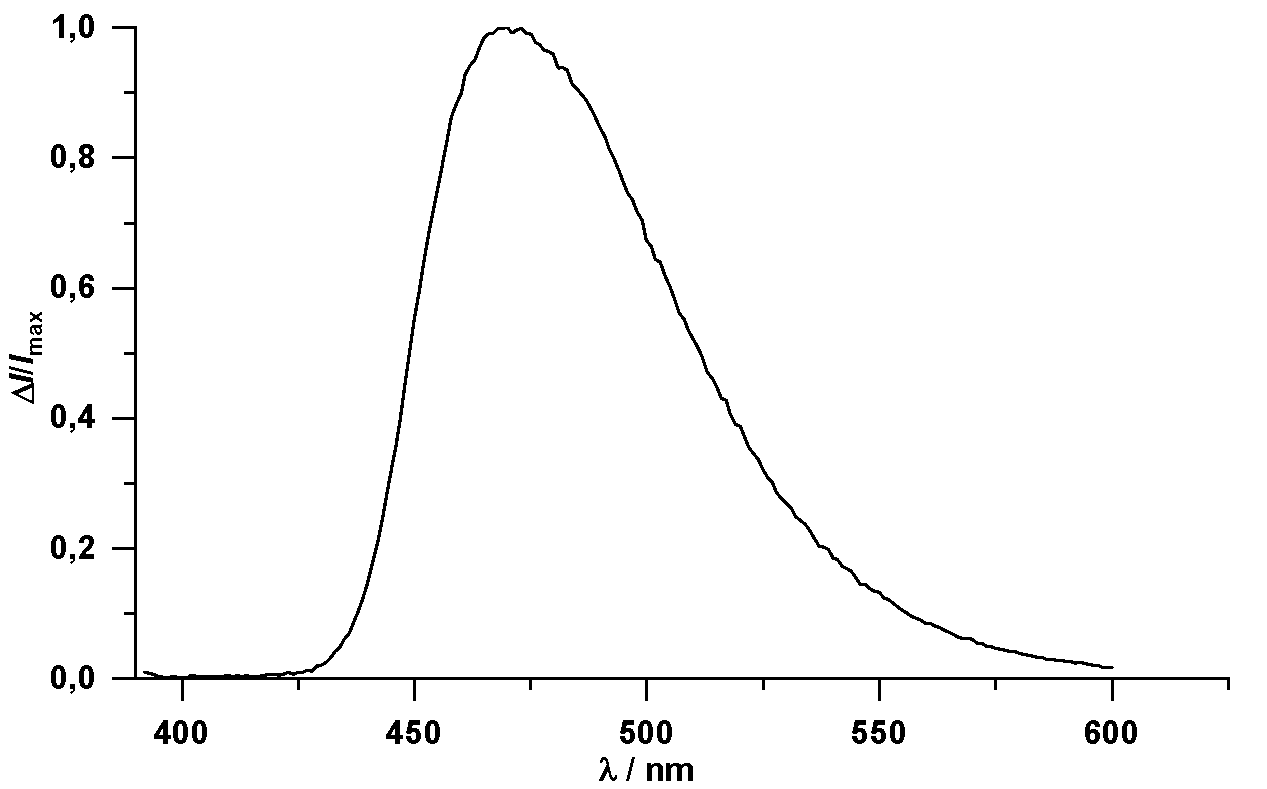


**Figure S10.** Fluorescence spectrum of **[27]helicenoid** with excitation at 338 nm in CH_2_Cl_2_.

5.1 Circularly Polarized Luminescence

**[27]helicenoid**

The spectra were recorded at ca. 5.5 × 10^-6^ M (for (*PM*) and (*MP*)) and were investigated in CH_2_Cl_2_. For CPL measurements, a fixed wavelength of 325 nm was provided by a Xenon Lamp source.


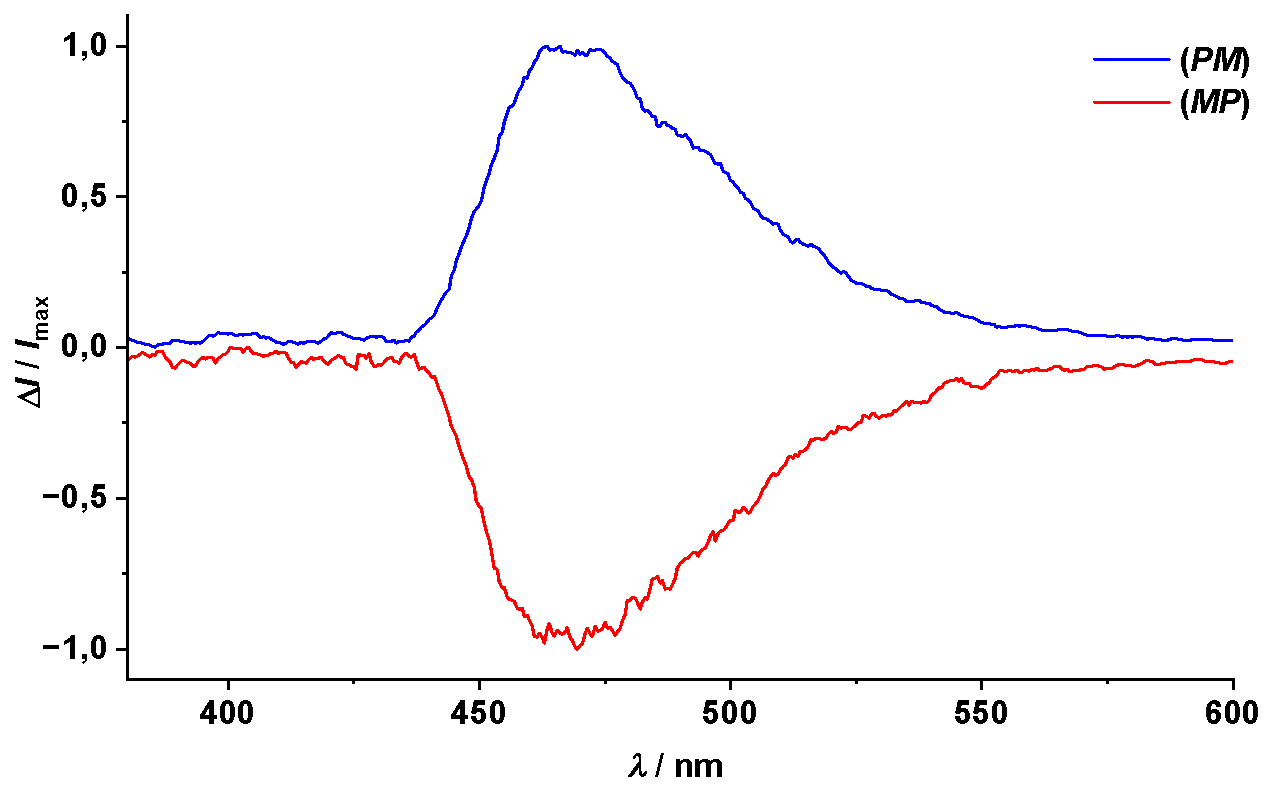


**Figure S11*.*** Circularly polarized luminescence spectra of **[27]helicenoid** in CH_2_Cl_2_ (λ_exc_ = 325 nm).


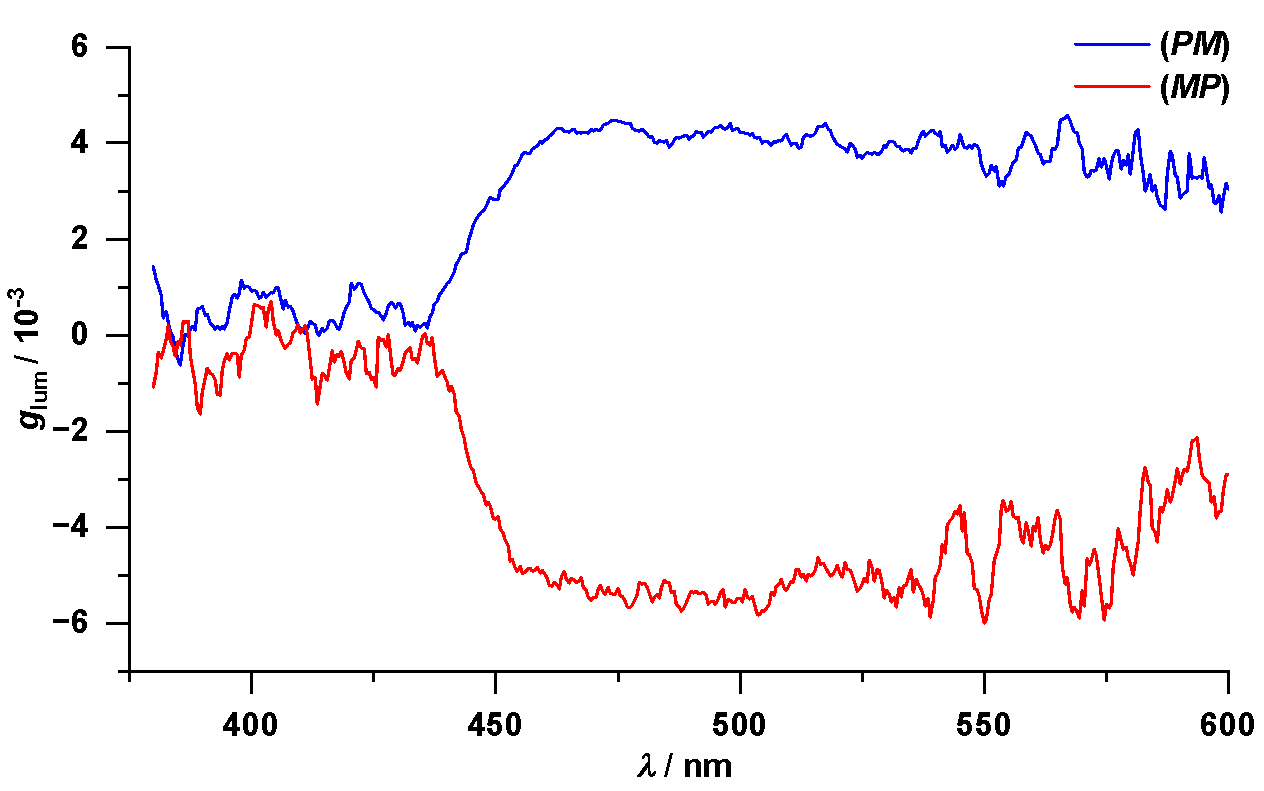


**Figure S12.** Dissymmetry factor (g_lum_) plot of circularly polarized luminescence spectra of **[27]helicenoid** in CH_2_Cl_2_ (λ_exc_ = 325 nm).

# Computational Details

The computational results are available on the following data repository: [ERDA link](https://erda.ku.dk/archives/5ba5ec39fabbeeb0a19a74cfb0866a39/published-archive.html).

For ease of access, input and output files are packed in a separate archive from the full results, which include wave functions and densities.

To make calculations on such a large structure viable, the geometry of **[27]helicenoid** was first simplified by substituting the propyl groups (on nitrogens) and *t*-butyl groups with methyl groups. This geometry was then optimized using the CAM-B3LYP^[15]^ functional with the D3BJ empirical dispersion correction^[16]^ and the def2-TZVP basis set^[17]^. Solvent effects were included implicitly through the CPCM model with parameters for dichloromethane. All calculations were performed using the ORCA 5.0.4^[1]^ software package and its default resolution-of-identity and chain of spheres (RIJCOSX)^[18-20]^ options. The computed solvated geometry is less distorted with the spiral turns stacked closer together than in the crystal structure (see Figure S3).

With such a large molecule, its ECD spectrum will have a rich structure, bound to be very sensitive to accurate rotatory strengths. For this reason, the 150 singlet electronic transitions were computed using full TD-DFT, without applying the Tam-Damcoff approximation (TDA-DFT)^[21]^, but keeping the aforementioned default RIJCOSX settings. An additional calculation of 30 singlet excitations with the larger, diffuse basis set ma-def2-TZVPP showed no significant difference, signaling basis set convergence.

For most of the analysis of ORCA results, we used the Multiwfn^[2]^ software package. Natural transition orbitals (NTOs) were computed using ORCA and visualized in Gabedit^[3]^. The spectrum was plotted using the default length gauge result, which matched the velocity gauge result.

To explore the chiroptical properties, we also modeled both the full and simplified (in the same manner as for **[27]helicenoid**) structures of **HO[13]OMe**, as well as a simplified [13]OMe+bridge structure (**[13]bridge**) which included the attached furan bridge, as well as part of the next helical turn (Figure S13). In the simplified **HO[13]OMe**, the transitions are redshifted by 0.02 to 0.10 eV, but stay qualitatively the same character to those in the full molecule, validating the approximation.

**Figure S13.** Simplified structures used for the examination of the optical properties of the (P)-**HO[13]OMe**, (P)-**[13]bridge** and (PM)-**[27]helicenoid** (from left to right).

The absolute configurations of respective enantiomers of **[27]helicenoid** were defined by comparison of the calculated and measured ECD spectra. The (*PM*) enantiomer, whose structure is shown in Figure S13, exhibits a positive Cotton effect near the absorption maximum. Empirical vibrational broadening of 0.50 eV was used and the calculated spectra were shifted by –0.50 eV. A good agreement of the alternating maxima in the experimental and calculated spectra was observed up to 350 nm, which was the reach of the computed spectrum.


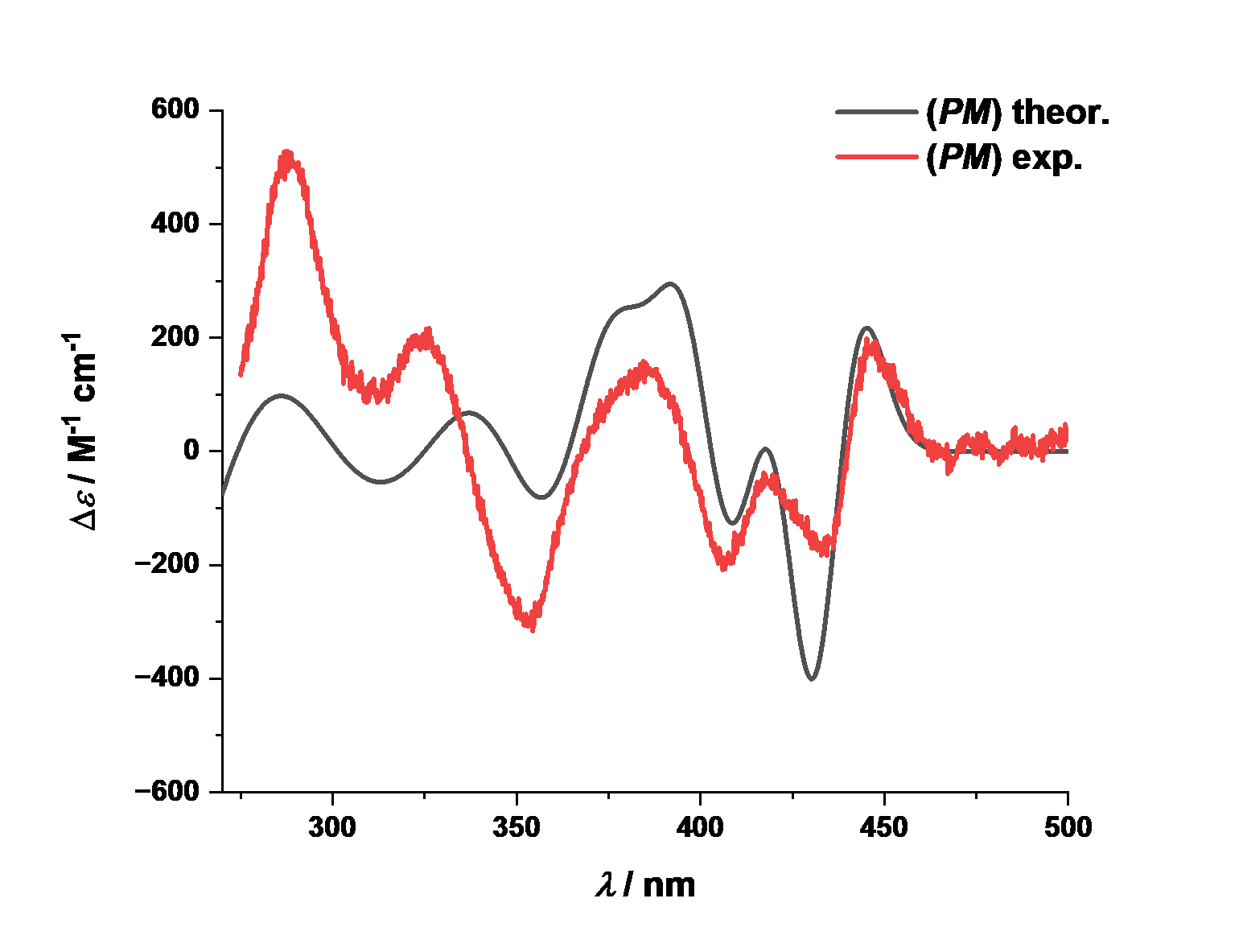


**Figure S14.** Experimental (red) and calculated (gray) ECD spectra of (PM)-**[27]helicenoid**.


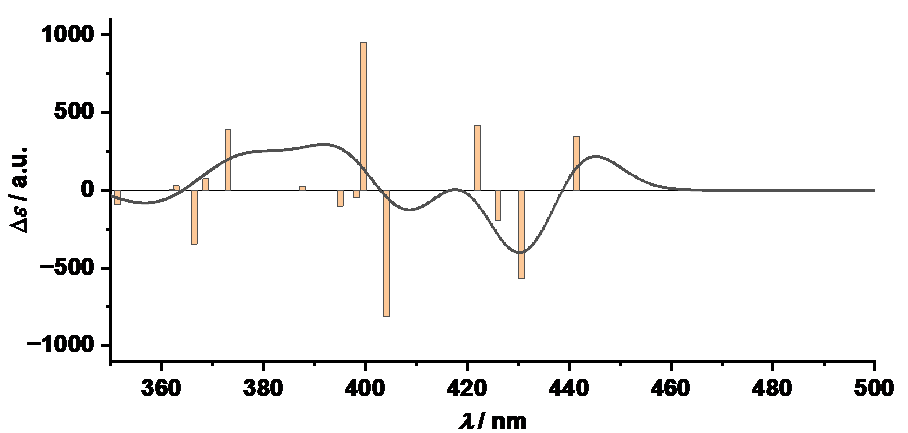


**Figure S15.** Calculated (black) full TD-DFT ECD spectrum of **[27]helicenoid** and its 150 component vertical transitions (orange bars) in CH_2_Cl_2_. Shifted by –0.50 eV and broadened with a 0.50 eV Gaussian.

The most striking difference between the **[27]helicenoid** and **HO[13]OMe** ECD spectra is the sudden alternation in the absolute magnitude of the optical rotation present in **[27]helicenoid**. We wished to identify whether this behavior is due to the inclusion of two helicene moieties of opposite configuration, forming the “telephone cord” structure, or simply due to extending the **HO[13]OMe** structure.

Over thirty electronic transitions make up the first four characteristic peaks of the spectrum in Figure S14, yet only six define the first four (two + and –) peaks as seen in Figure S15. They also have the strongest calculated rotatory strengths, which is why we will focus on them. First off, the computed NTOs (Figure S16) of **[27]helicenoid** show that the electronic transitions of interest are, as expected, delocalized π-π* excitations. However, they only delocalize over one-half of the structure – alternating with which half of the system they cover as the energy increases.

However, the NTOs of **[27]helicenoid** reach into the central bridge area, meaning that even if the structure behaves optically as two (*M*, *P*) or (*P*, *M*) helicenes, the effect of slightly extending the conjugation length cannot be disregarded. As presented in Table S2, the **[13]bridge** structure, which includes the furan bridge, as well as the next turn of the helicene with which it is bridged, already stabilizes the S_1_ state by around 630 cm^-1^ (0.08 eV) matching the energy of the lowest transition in the **[27]helicenoid**. This shift is a similar 0.06 eV when comparing to the simplified **HO[13]OMe**. This redshift becomes much smaller for the optically bright states, explaining the similarity of the **[13]** and **[27]helicenoid** UV-VIS spectra.


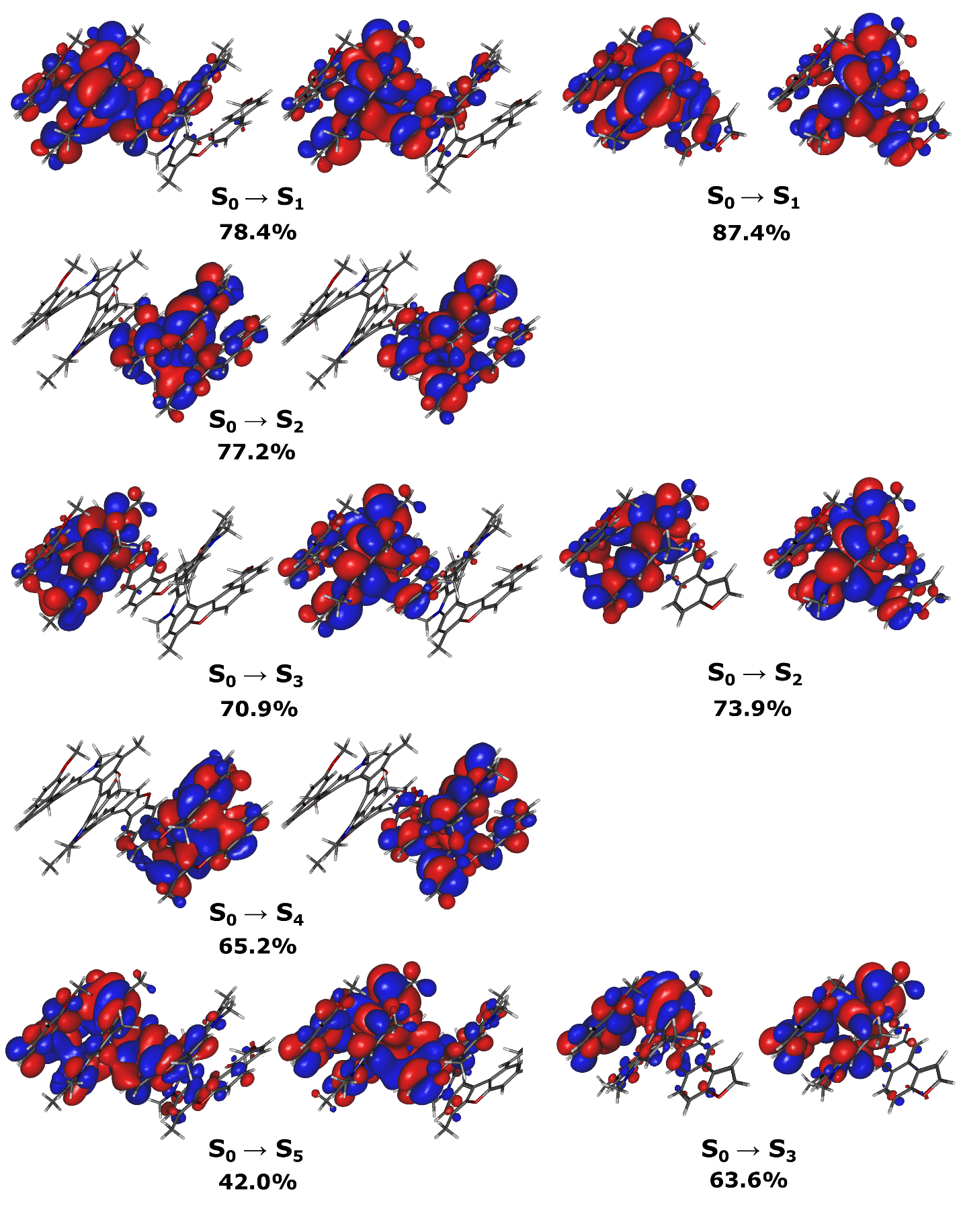


**Figure S16.** NTOs of the relevant transitions of the **[27]helicenoid** (left) and **[13]bridge** (right) with their eigenvalues in percentages calculated in CH_2_Cl_2_ at TD-CAM-B3LYP/def2-TZVP level of theory. 90% of electronic density shown.

Interestingly, the rotatory strength of the optically brightest transitions in **[27]helicenoid** (S_5_, S_6_, S_8_) are greatly increased in **[27]helicenoid** compared to those in the smaller structures. Additionally, the associated NTOs are not well described with a single hole and electron orbital pair, with significant contributions of NTOs that are fully delocalized over the entire structure. While effects of the delocalization are diminished, they are not completely absent.

In the **HO[13]OMe** results, S_1_ and S_2_ also have different signs of rotatory strength, but they are of very similar energy. This results in the first peak having a lower signal, but would not show up in the structure of the spectrum.

Results for the **[27]helicenoid** structure are listed in Table S2 and it shows that the initial structure of the spectrum matches well an interlaced pattern of opposite rotating **HO[13]OMe** transitions, with one set stabilized by the presence of the bridge. The energy shift of S_1_ is already very well reproduced even with a single additional turn of the helicene past the furan bridge, which can also be seen in the associated NTOs of **[27]helicenoid**, which do not reach past it.

Finally, the S_1_ minimum was obtained for **[27]helicenoid** and **HO[13]OMe** in order to study the computed *g*_abs_ and *g*_lum_ values. The optimization was performed at the same level of theory with the TDA approximation applied. This also allows us to check the energy of the 0-0 transition for S_1_ of **[27]helicenoid**, which at 24725 cm^-1^, is around 0.25 eV lower than the vertical transition, which accounts for half of the redshift required to match the experimental spectrum.

**Table S2.** Properties of the lowest energy singlet excitations of the three simplified structures as obtained from a full TD-DFT calculation.

| **[27]helicenoid** (simplified, *PM*) - 183 atoms | | | | |
| --- | --- | --- | --- | --- |
| **State** | ***E* / cm^-1^** | ***R* / 1e40*cgs** | ***f_osc_*** | **Moeity (NTO)** |
| S_1_ | 26684.0 | **347.2** | 0.135 | P |
| S_2_ | 27259.4 | **-566.2** | 0.045 | M |
| S_3_ | 27506.7 | -191.4 | 0.071 | P |
| S_4_ | 27735.1 | 418.1 | 0.103 | M |
| S_5_ | 28777.8 | **-811.4** | **0.476** | P^d^ |
| S_6_ | 29055.4 | **950.6** | **0.302** | P^d^ |
| S_7_ | 29143.2 | -46.7 | 0.041 | P |
| S_8_ | 29346.9 | -102.0 | **0.419** | M^d^ |

^d^ These transitions have higher contributions from a second NTO pair which is delocalized

over the entire structure.

| **[13]bridge** (simplified, *P*) - 111 atoms | | | |
| --- | --- | --- | --- |
| **State** | ***E* / cm^-1^** | ***R* / 1e40*cgs** | ***f_osc_*** |
| S_1_ | 26862.1 | **518.7** | 0.093 |
| S_2_ | 27535.1 | -206.5 | 0.087 |
| S_3_ | 29189.3 | -175.0 | **0.523** |
| S_4_ | 29536.2 | -31.7 | 0.050 |
| S_5_ | 30890.0 | 229.9 | 0.068 |
| S_6_ | 31280.2 | 228.6 | **0.209** |

| **HO[13]OMe** - 142 atoms | | | | **HO[13]OMe** (simplified, *P*) - 97 atoms | | |
| --- | --- | --- | --- | --- | --- | --- |
| **State** | ***E* / cm^-1^** | ***R* / 1e40*cgs** | ***f_osc_*** | ***E* / cm^-1^** | ***R* / 1e40*cgs** | ***f_osc_*** |
| S_1_ | 27493.5 | **682.4** | 0.140 | 27315.6 | **783.5** | 0.119 |
| S_2_ | 27714.1 | -268.6 | 0.109 | 27668.9 | -343.1 | 0.093 |
| S_3_ | 29170.9 | -80.4 | **0.716** | 29063.1 | -66.5 | **0.676** |
| S_4_ | 30464.6 | -284.8 | 0.150 | 29648.7 | -140.4 | 0.040 |
| S_5_ | 31908.3 | 486.0 | **0.405** | 31222.5 | 360.4 | **0.384** |

**Table S3.** Comparison of experimental and calculated (with full TD-DFT) *g*_abs_ and *g*_lum_ values of the simplified **[27]helicenoid** and the full structure of **HO[13]OMe**.

| **Structure** | **E (cm^-1^) of S_0_-S_1_ transition** | **T2 (au**2)** | **R (1e40*cgs)** | **Calc. *g*_lum_ value (10^-3^)** | **Exp. *g*_lum_ value** |
| --- | --- | --- | --- | --- | --- |
| **[27]helicenoid** at S_1_ minimum | 22027.8 | 1.64290 | 408.78285 | **15.41** | **5.5** |
| **HO[13]OMe** (full) at S_1_ minimum | 23407.6 | 2.68118 | 584.62283 | **13.50** | **4.2** |
| **Structure** | **E (cm^-1^) of S_0_-S_1_ transition** | **T2 (au**2)** | **R (1e40*cgs)** | **Calc. *g*_lum_ value (10^-3^)** | **Exp. *g*_lum_ value** |
| **[27]helicenoid** at S_1_ minimum | 22027.8 | 1.64290 | 408.78285 | **15.41** | **5.5** |
| **HO[13]OMe** (full) at S_1_ minimum | 23407.6 | 2.68118 | 584.62283 | **13.50** | **4.2** |

# NMR Spectra

**Figure S17.** ^1^H-NMR (500 MHz, CD_2_Cl_2_, 298 K) spectrum of **[27]helicenoid**.


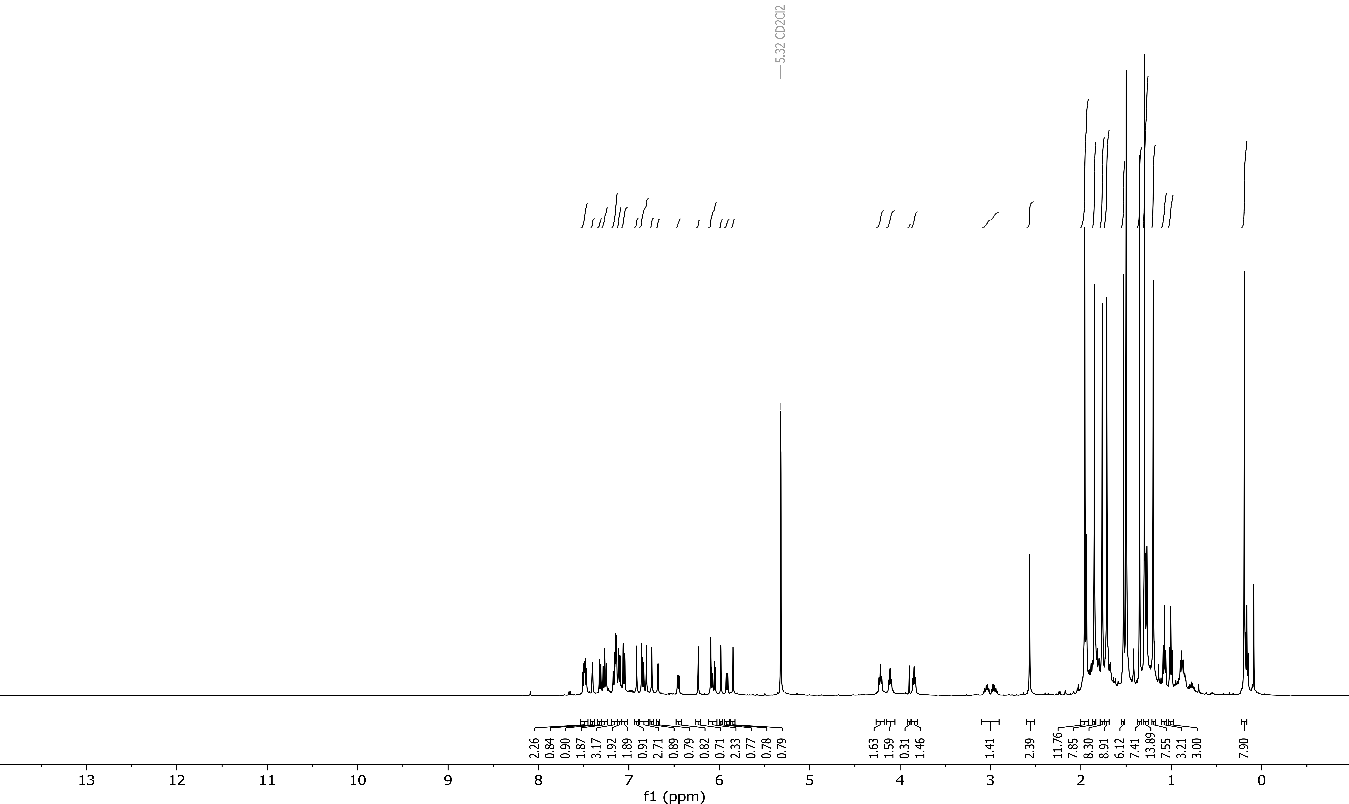

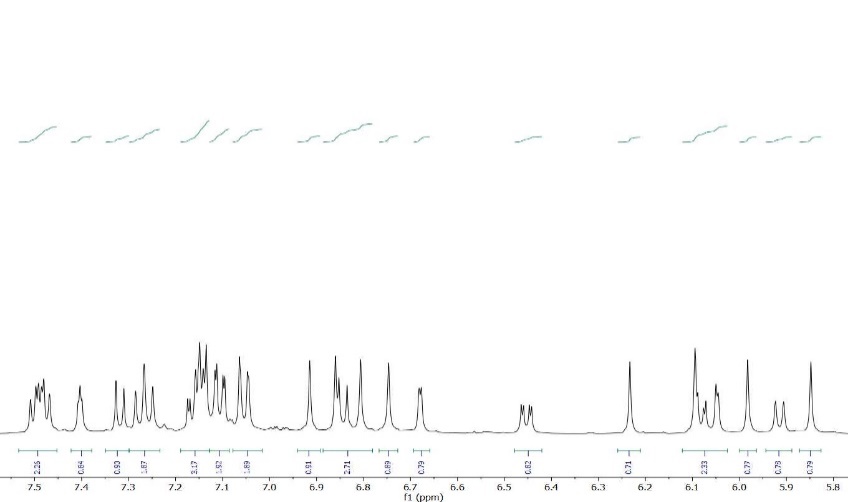


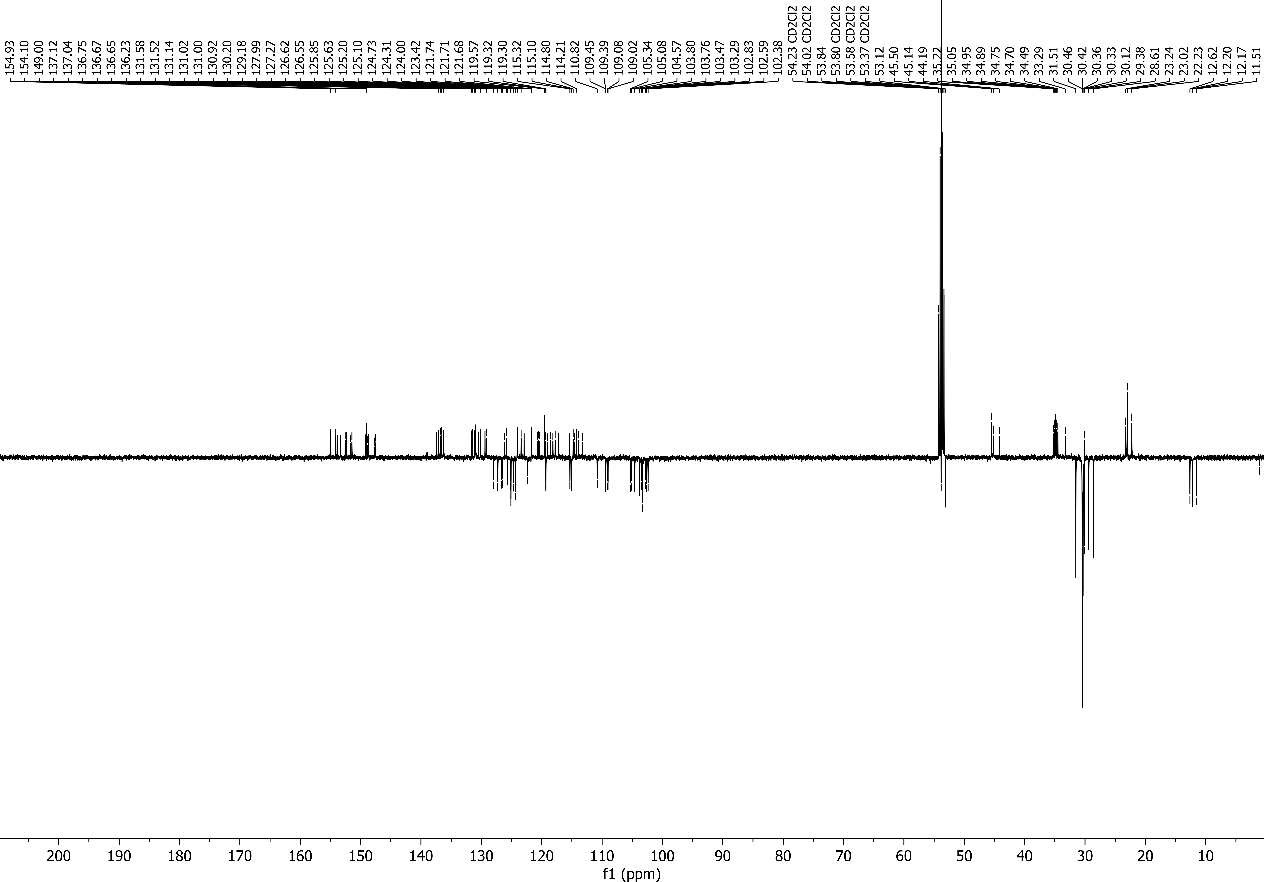


**Figure S18.** ^13^C-NMR (500 MHz, CD_2_Cl_2_, 298 K) spectrum of **[27]helicenoid**.


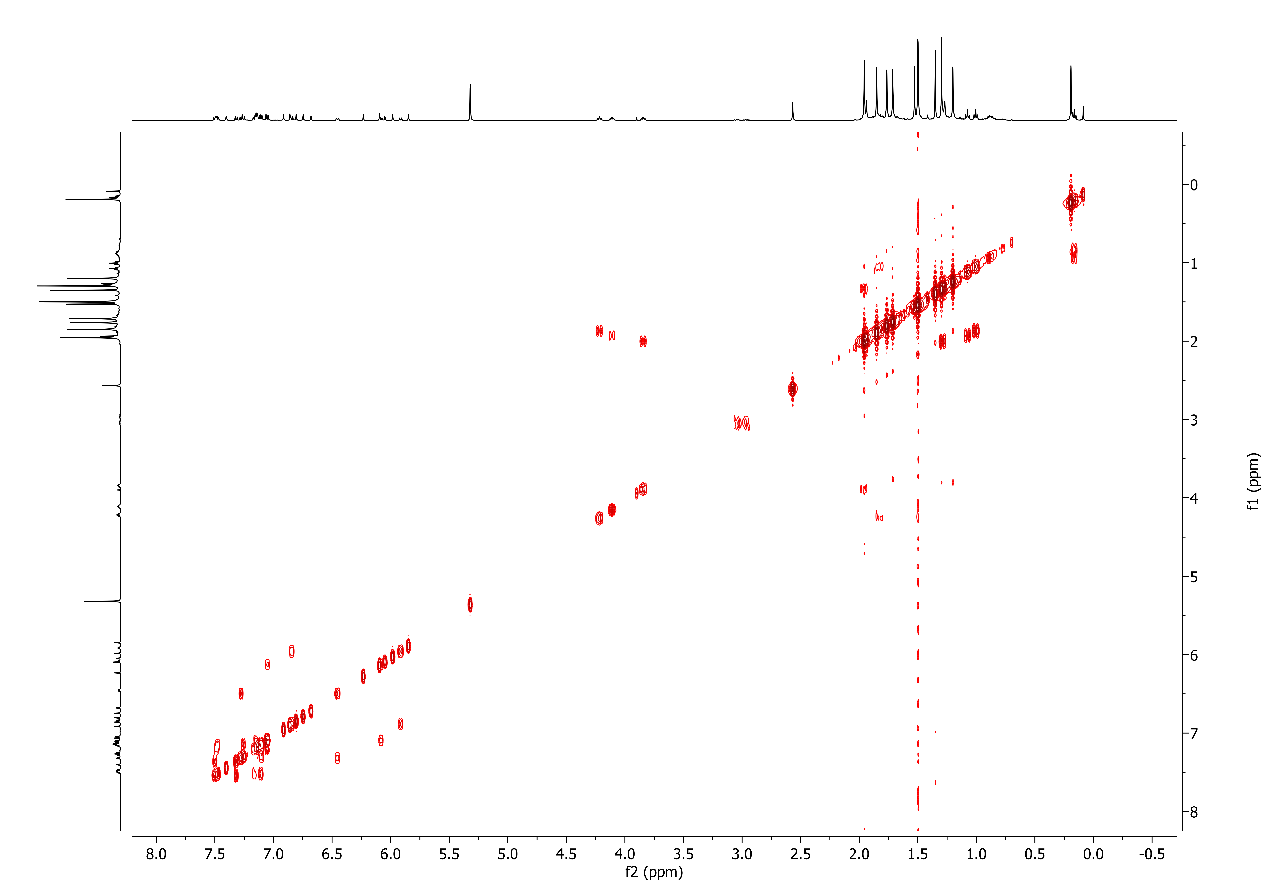


**Figure S19.** ^1^H–^1^H COSY (500 MHz, CD_2_Cl_2_, 298 K) spectrum of **[27]helicenoid**.


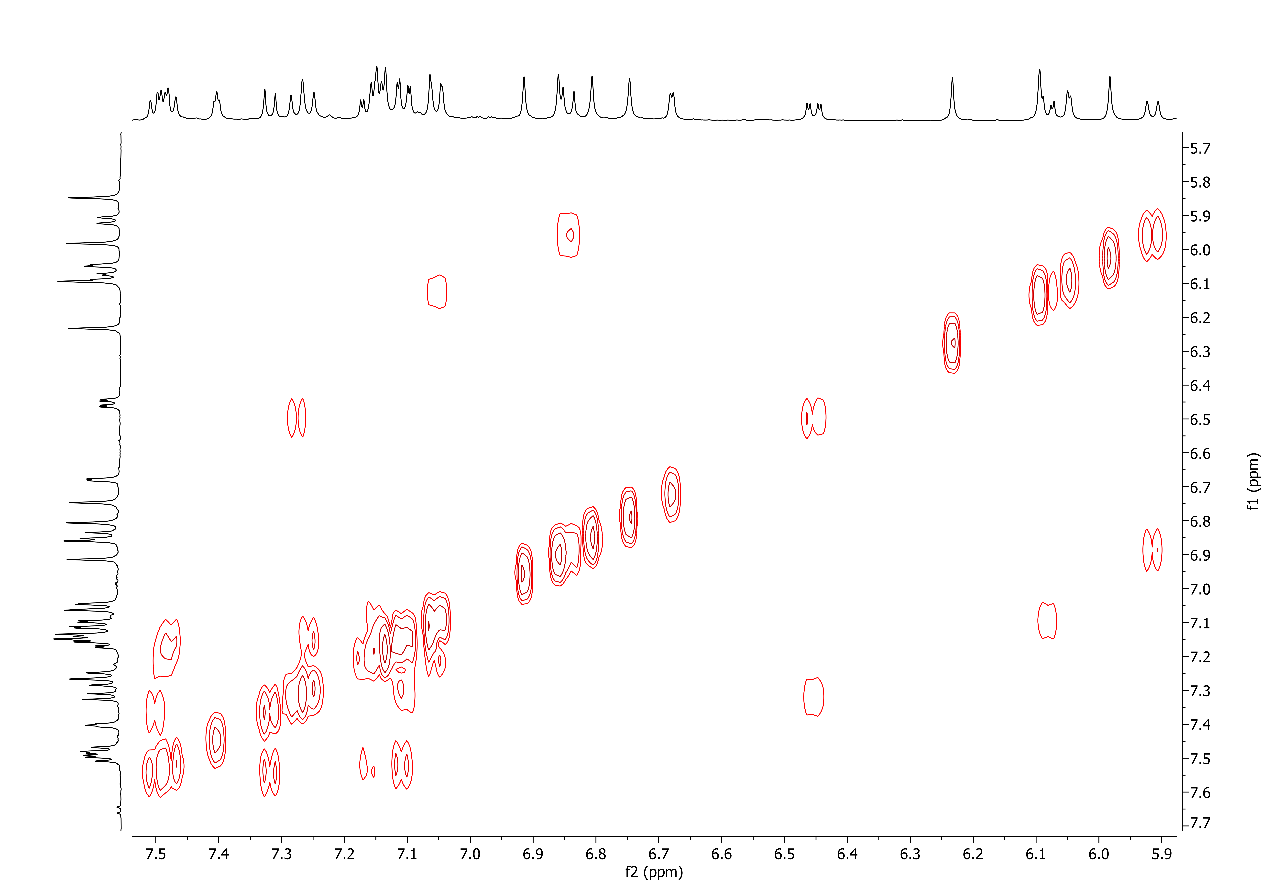


**Figure S20.** Aromatic region of the ^1^H–^1^H COSY spectrum of **[27]helicenoid** (500 MHz, CD_2_Cl_2_, 298 K).


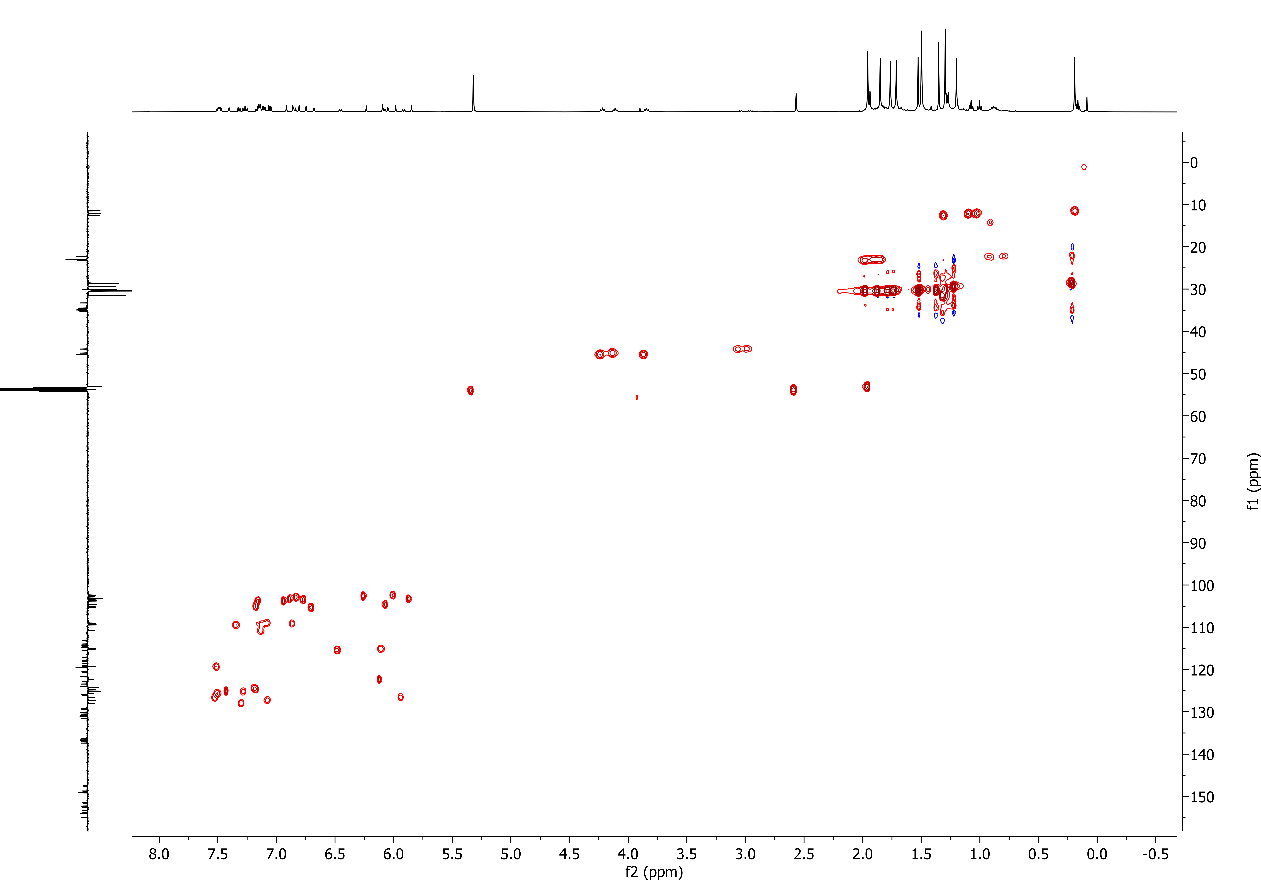


**Figure S21.** ^1^H–^13^C HSQC spectrum (500 MHz, CD_2_Cl_2_, 298 K) of **[27]helicenoid**.


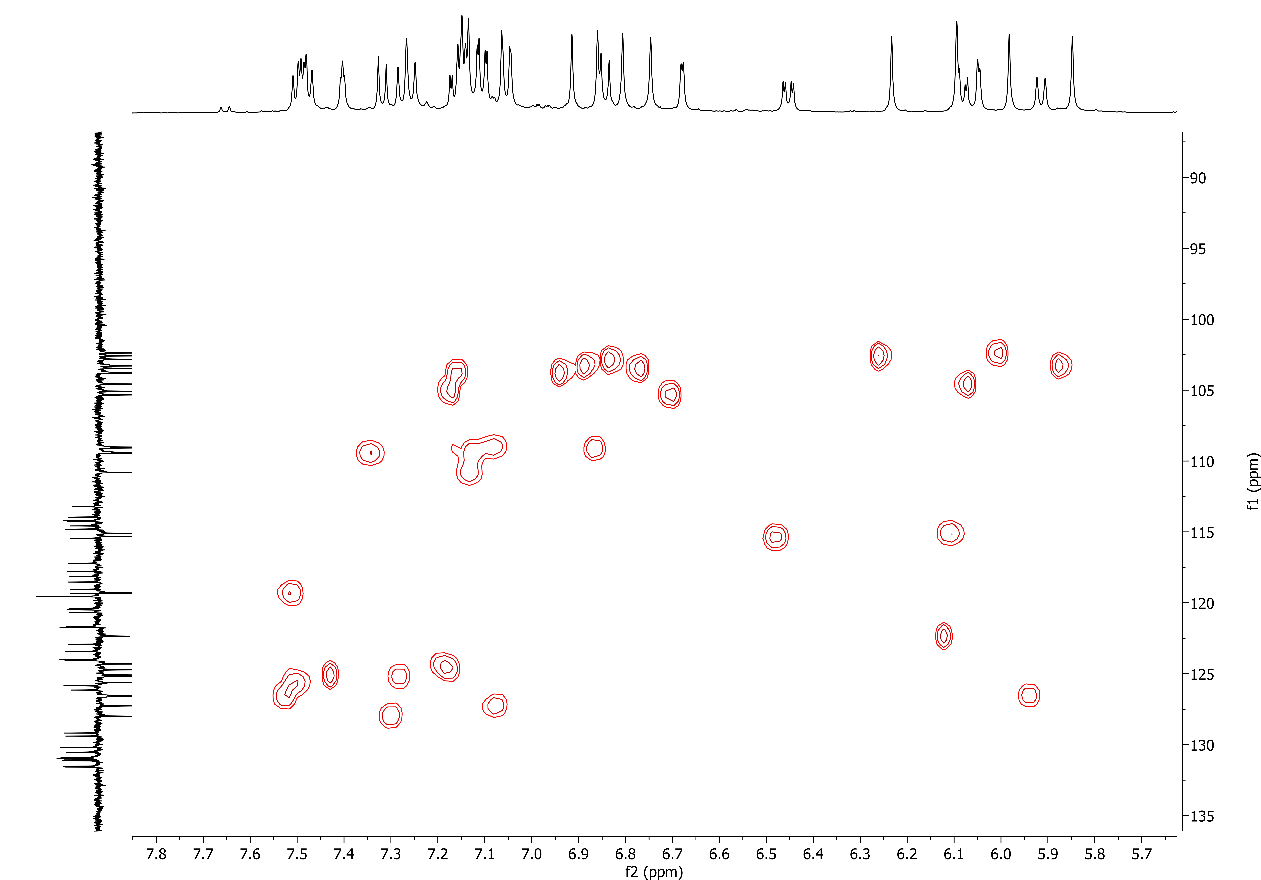


**Figure S22.** Aromatic region of the ^1^H–^13^C HSQC spectrum (500 MHz, CD_2_Cl_2_, 298 K) of **[27]helicenoid**.

# HRMS Spectra


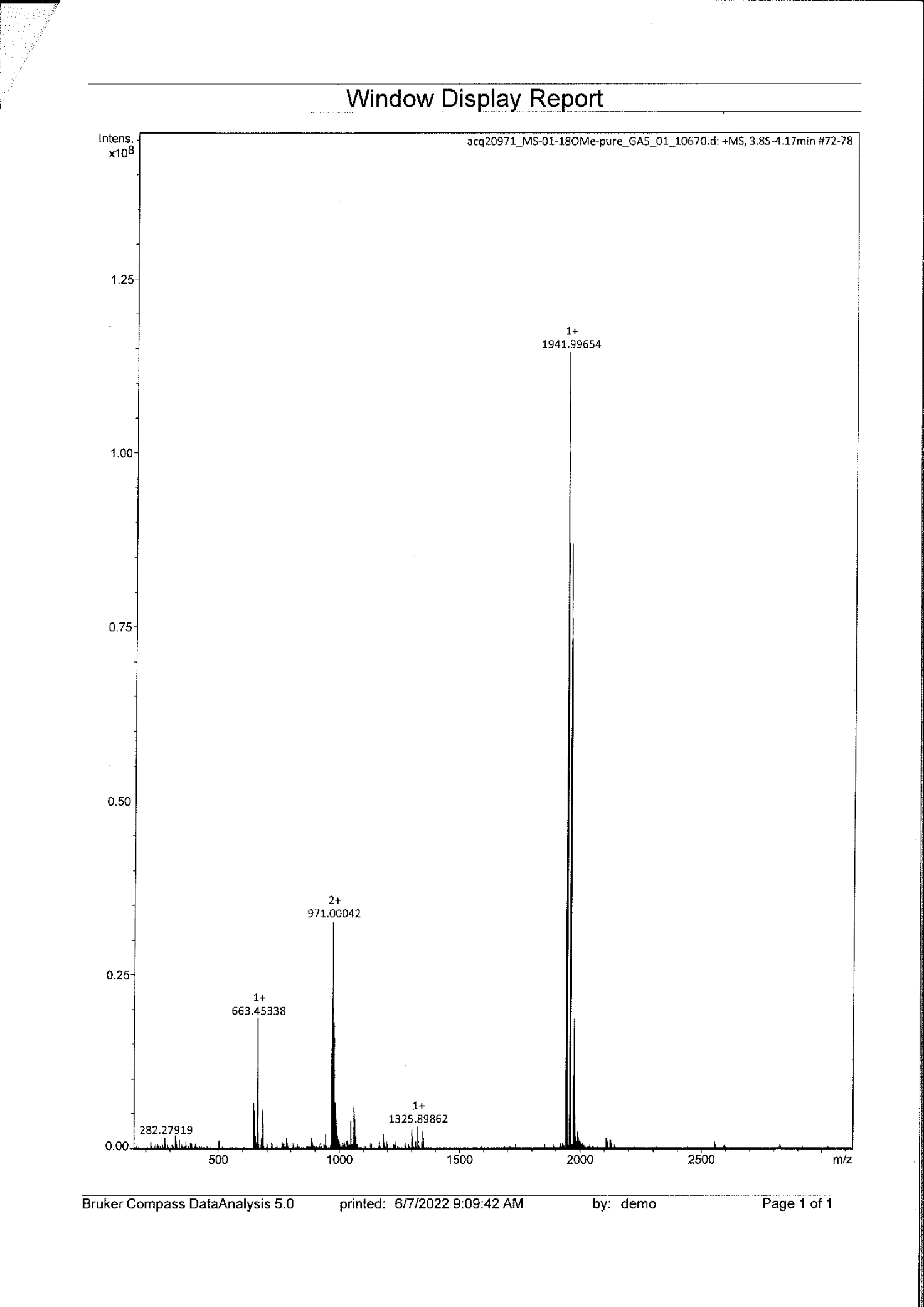


**Figure S23.** HRMS (MALDI‑TOF) spectrum of **[27]helicenoid** [M]^+^.

|  |
| --- |
| 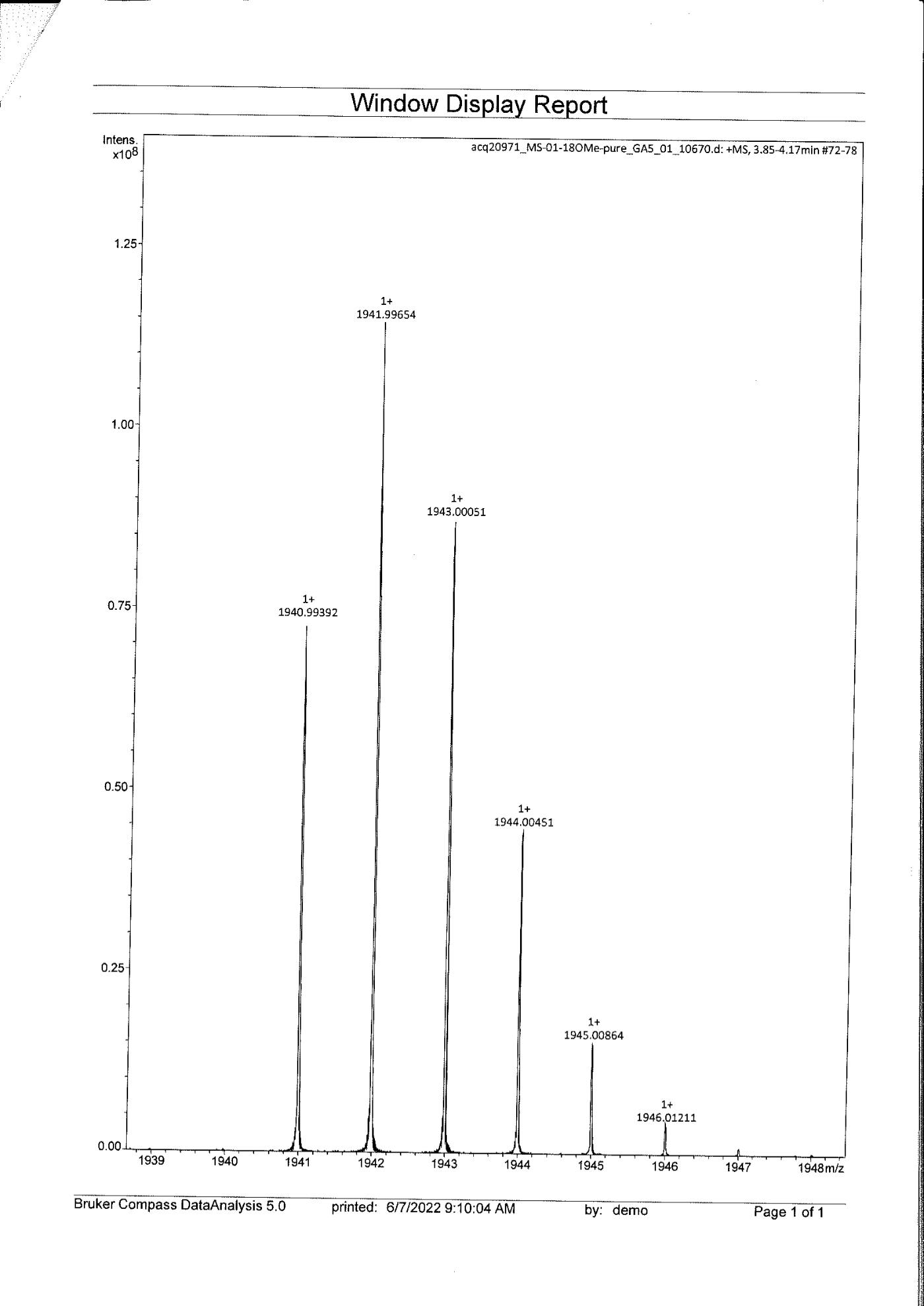  **Figure S24.** HRMS MALDI‑TOF (Zoomed-In View): Isotopic Distribution of **[27]helicenoid** [M]^+^ Ion. |
|  |
| 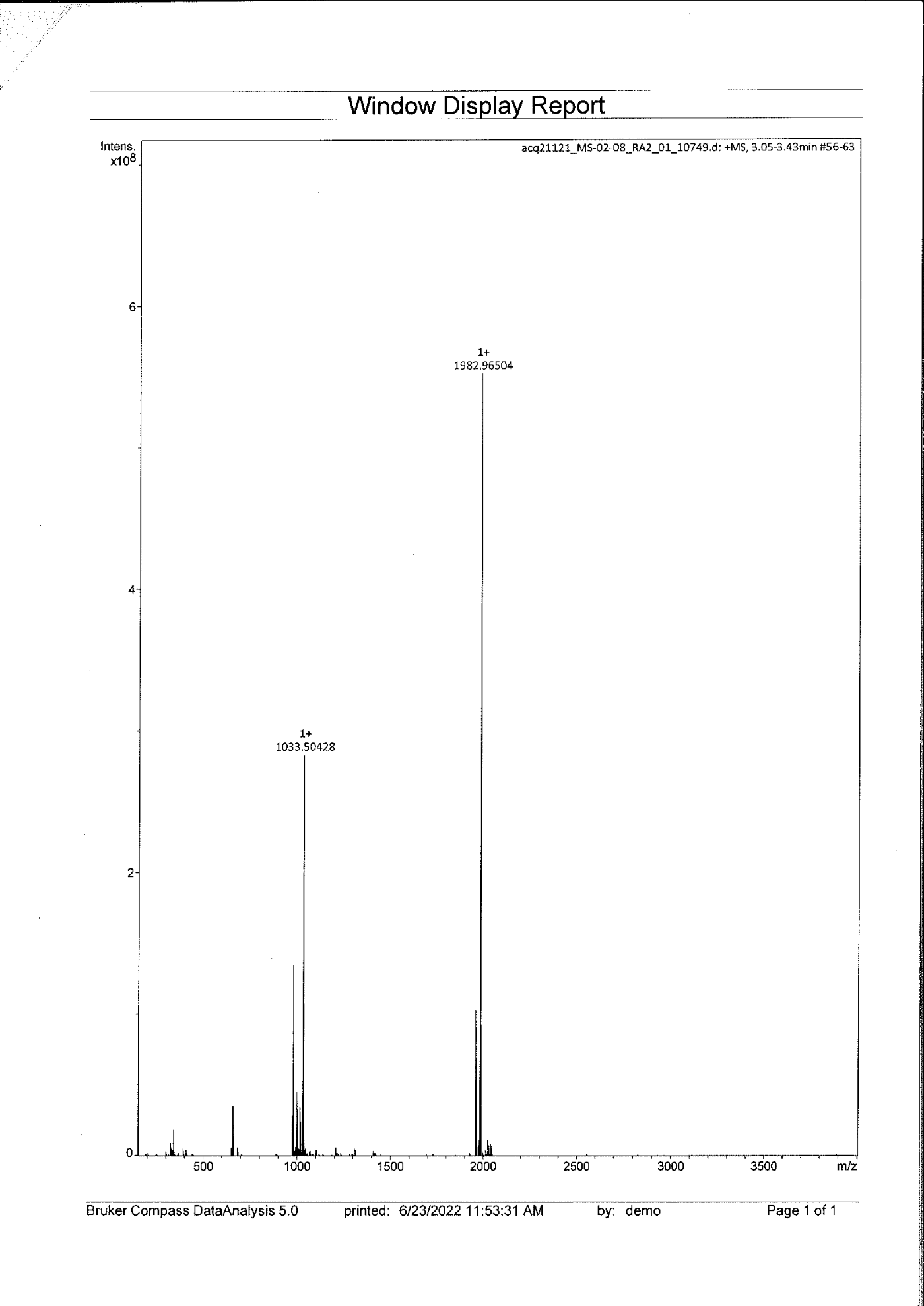  **Figure S25.** HRMS (MALDI‑TOF) spectrum of **bi[13]** [M+Na]^+^. |
| 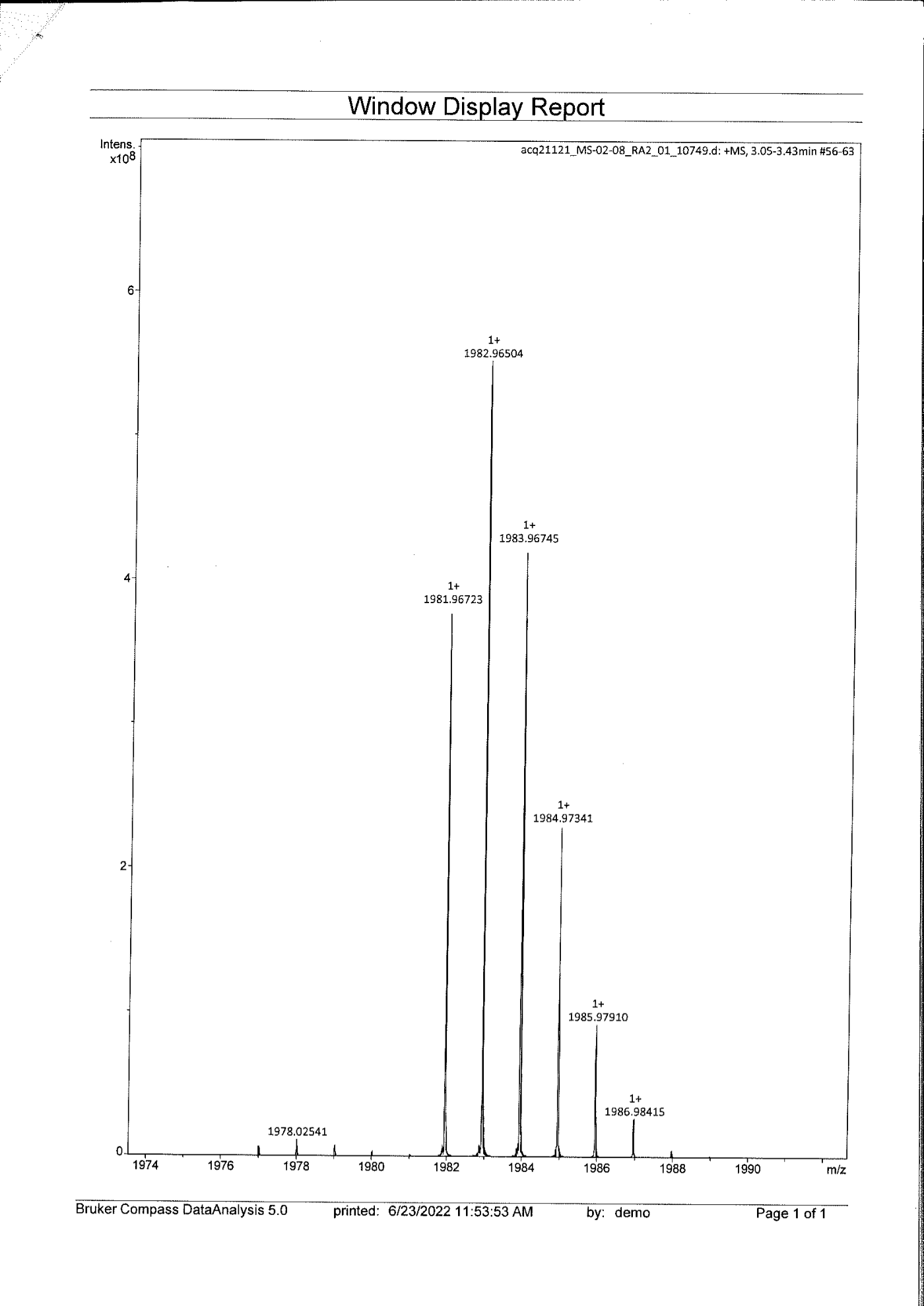  **Figure S26.** HRMS (MALDI‑TOF) (Zoomed-In View): Isotopic Distribution of **bi[13]** [M+Na]^+^ Ion. |

# References

[1] F. Neese, *Wiley Interdiscip. Rev. Comput. Mol. Sci.* **2022**, *12*.

[2] T. Lu, F. Chen, *J. Comput. Chem.* **2012**, *33*, 580.

[3] A.-R. Allouche, *J. Comput. Chem.* **2011**, *32*, 174.

[4] *SAINT (Version 8.40B): Area Detector Control and Integration Software*, Bruker AXS Inc., Madison, Wisconsin (USA), **2019**.

[5] L. Krause, R. Herbst-Irmer, G. M. Sheldrick, D. Stalke, *J. Appl. Crystallogr* **2015**, *48*, 3.

[6] G. M. Sheldrick, *Acta Cryst.* **2015**, *71*, 3.

[7] G. M. Sheldrick, *Acta Crystallogr. Sect. C Struct. Chem.* **2015**, *71*, 3.

[8] C. B. Hübschle, G. M. Sheldrick, B. Dittrich, *J. Appl. Crystallogr.* **2011**, *44*, 1281.

[9] O. V. Dolomanov, L. J. Bourhis, R. J. Gildea, J. A. K. Howard, H. Puschmann, *J. Appl. Cryst.* **2009**, *42*, 339.

[10] Rigaku Corporation, *CrysAlisPro*, Rigaku Oxford Diffraction Ltd, Wroclaw, Poland, **2024**.

[11] I. Usón, G. M. Sheldrick, *Curr. Opin. Struct. Biol.* **1999**, *9*, 643.

[12] L.-M. Peng, *Micron* **1999**, *30*, 625.

[13] C. F. Macrae, I. Sovago, S. J. Cottrell, P. T. A. Galek, P. McCabe, E. Pidcock, M. Platings, G. P. Shields, J. S. Stevens, M. Towler et al., *J. Appl. Cryst.* **2020**, *53*, 226.

[14] S. K. Pedersen, K. Eriksen, M. Pittelkow, *Angew. Chem. Int. Ed.* **2019**, *58*, 18419.

[15] T. Yanai, D. P. Tew, N. C. Handy, *Chem. Phys. Lett.* **2004**, *393*, 51.

[16] S. Grimme, J. Antony, S. Ehrlich, H. Krieg, *J. Chem. Phys.* **2010**, *132*, 154104.

[17] F. Weigend, R. Ahlrichs, *Phys. Chem. Chem. Phys.* **2005**, *7*, 3297.

[18] F. Neese, G. Olbrich, *Chem. Phys. Lett.* **2002**, *362*, 170.

[19] F. Neese, *J. Comp. Chem.* **2003**, *24*, 1740.

[20] F. Neese, F. Wennmohs, A. Hansen, U. Becker, *Chem. Phys.* **2009**, *356*, 98.

[21] S. Hirata, M. Head-Gordon, *Chem. Phys. Lett.* **1999**, *314*, 291.
